# The psychometric network of individual flourishing across nationally representative samples from 22 countries

**DOI:** 10.1038/s41598-025-15016-6

**Published:** 2025-08-18

**Authors:** Michela Zambelli, Dwight C. K. Tse, Richard G. Cowden, Jan Höltge, Byron R. Johnson, R. Noah Padgett, Tyler J. VanderWeele

**Affiliations:** 1 Università Cattolica del Sacro Cuore, Milan, Italy; 2 University of Strathclyde, Glasgow, Scotland, UK; 3 Harvard University, Cambridge, MA USA; 4 Dalhousie University, Halifax, NS Canada; 5 Baylor University, Waco, TX USA

**Keywords:** Global flourishing study, Psychometric network analysis, Meta-analytic gaussian network aggregation, Cross-country, Psychology, Human behaviour

## Abstract

To effectively promote human flourishing, it is important to understand how the different dimensions of flourishing might be related to one another in different sociocultural contexts. Applying a systems perspective to flourishing, this study uses nationally representative survey cross-sectional data from 22 geographically and culturally diverse countries included in the Global Flourishing Study (*N* = 202,898) to explore the interrelatedness of the components of individual flourishing captured by the Secure Flourish Measure. A meta-analytic gaussian network aggregation (MAGNA) model was applied to investigate similarities and differences among the interrelations of individual flourishing components across countries. Results revealed a network of mostly positive interrelations among the 12 components, although there was substantial heterogeneity in the strength of associations, especially between life satisfaction, happiness, and mental health. Understanding cross-country differences in light of socio-contextual peculiarities will be crucial for informing the development of targeted interventions to promote flourishing.

## Introduction

Human flourishing refers to a state of complete well-being, such that “all aspects of a person’s life are good”^1^, p. 3). In line with the United Nation’s Sustainable Development Goals (SDGs) of good health and well-being, many countries have incorporated indices of well-being into their policymaking considerations^2,3^. Although various theories of human flourishing have been proposed, most come to a consensus that the concept is multidimensional. For example, Keyes^4^ described flourishing as the pinnacle of mental health based on three broad dimensions of emotional, psychological, and social well-being, whereas Seligman’s^5^ PERMA model identifies positive emotion, engagement, relationships, meaning, and accomplishment as the foundation for a flourishing life. One prominent model of individual flourishing (distinct from the broader concept of human flourishing that includes the context of healthy communities enabling a good life,see^1,6^) that has emerged in recent years prioritizes five core domains—(1) happiness & life satisfaction, (2) mental & physical health, (3) meaning & purpose, (4) character & virtue, and (5) close social relationships—which are seen as near-universally desired and ends in themselves^7,^^8^). A sixth domain, financial & material stability, is considered a means of sustaining the five core domains over time. Although this six-domain model should not be viewed as providing a fully exhaustive account of individual flourishing, it addresses some gaps in existing models of flourishing. For instance, some prior research has found that physical health is considered one of the most important domains of individual flourishing^9,10^, yet it is not part of Seligman’s^5^ PERMA model.

Ongoing validation work of the Secure Flourish Measure (SFM^7^;,see also Table 1 for its items), a 12-item self-report measure that is based on this six-domain individual flourishing model, has shown promising results in nationally representative samples^11–14^, in specific life domains, such as workplaces^11,12,14^,Węziak-Białowolska et al., 2019a), and across different cultures^15,16^^17^,). Existing evidence supports the validity and wide applicability of the SFM in various populations.

**Table 1:** The Secure Flourish Measure (SFM).

	**Flourishing dimensions**	**Items wording**	**Items acronym**
1	Happiness & Life satisfaction	Overall, how satisfied are you with life as a whole these days?	LS = life satisfaction
		In general, how happy or unhappy do you usually feel?	H = happiness
2	Mental & Physical health	In general, how would you rate your physical health?	MH = mental health
		How would you rate your overall mental health?	PH = physical health
3	Meaning & Purpose	Overall, to what extent do you feel the things you do in your life are worthwhile?	W = life worthwhile
		I understand my purpose in life	SP = sense of purpose
4	Character & Virtue	I always act to promote good in all circumstances, even in difficult and challenging situations	PG = promote good
		I am always able to give up some happiness now for greater happiness later	GU = give up happiness
5	Close social relationships	I am content with my friendships and relationships	C = content with relationships
		My relationships are as satisfying as I would want them to be	SR = satisfying relationships
6	Financial & Material stability	How often do you worry about being able to meet normal monthly living expenses?	E = worry about expenses
		How often do you worry about safety, food, or housing?	WS = worry about safety

*Notes*. In past studies, results were typically reported separately for each domain by taking an average of the two items, with higher scores indicating greater individual flourishing in that domain. Averages can also be taken across the domains as a crude assessment of individual flourishing. In this study, however, we uesd items raw scores for all our subsequent analyses.

Nevertheless, the concept of human flourishing has been subject to continuous debate. Notable differences exist between its definitions in various academic disciplines: whereas some theorists reduce human flourishing to a multifaceted psychological state, others highlight its dynamic and complex nature encompassing a person’s optimal development within their environment^1,16,18^. One criticism underscores that an individual-focused approach to human flourishing lacks awareness of the impacts of structure, power, and history on health and well-being^19^. As humans grow and develop in various sociocultural contexts, this criticism poses a question as to whether a top-down, etic approach that focuses primarily on internal psychological states can effectively capture the essence of human flourishing for most, if not all, individuals worldwide.

Past studies have provided preliminary insights into the cross-cultural generalizability of the six-domain individual flourishing model. In one study, cross-sectional survey data from over 7,000 emerging adults from 10 countries spanning five continents were used to estimate the psychometric networks of individual flourishing^16^. This analytic method resonates with socioecological theory^20^, which asserts that individual flourishing can only be fully understand by considering higher-level contextual factors (e.g., culture) that characterize the environment in which a person is embedded. A systems perspective of human flourishing acknowledges the interdependence of different dimensions of human life, such that the constituents of flourishing are theorized to influence each other cross-sectionally and longitudinally. Studies have increasingly adopted such a perspective to understand well-being and ill-being (e.g^21–25^.,). This multisystemic network approach is especially compatible with the study of human flourishing, which comprises complex dynamics among various aspects of a good life and the contextual factors of the environment^1,19^. Höltge et al.’s^16^ findings revealed country-specific networks with unique patterns of interconnections between individual flourishing domains, but there were also substantial similarities across countries, suggesting that the individual flourishing domains were indeed interrelated in the sampled countries.

While Höltge et al.’s^16^ findings represent an important step forward in our understanding of the interrelations among different domains of individual flourishing, there were several methodological limitations of their study. First, the data were collected from convenience samples of emerging adults (18–29 years) in a few non-strategically selected countries. Therefore, it is unclear whether their findings are generalizable to the adult populations from which they were drawn, and further work is needed to determine whether their findings are transportable to distinct cultural contexts that were not represented in their data. These limitations may be mitigated by including nationally representative samples from a wider range of geographically and culturally diverse countries. Second, for practical reasons Höltge et al.^16^ examined the network configuration of individual flourishing by aggregating the 12 SFM components into their six domains (i.e., happiness & life satisfaction, mental & physical health, meaning & purpose, character & virtue, close social relationships, and financial & material stability). However, because each item included in the SFM represents a distinct component of individual flourishing^7^, aggregating components by domain may mask useful insights about the interrelatedness between different aspects of individual flourishing. For example, although meaning in life and sense of purpose are highly correlated, given the nuanced differences between the concepts (e.g., purpose being more goal-oriented), they may differ in their strength of associations with other components of individual flourishing.

### The present study

To address these gaps, the present study aims to examine the psychometric network of individual flourishing in nationally representative samples from 22 countries (representing approximately half of the world’s population) included the first wave of the Global Flourishing Study (GFS^26^;). Specifically, we decided to explore the unique contribution of the 12 individual flourishing components included in the SFM proposed by VanderWeele^7^ and investigate whether the interrelations between constituents of individual flourishing are generalizable across countries. Despite the study nature being exploratory rather than making causal inferences, we anticipate (a) to observe positive interrelations between the 12 components, confirming their individual contribution to individual flourishing across countries,and (b) that the meta-analytic approach would highlight heterogeneity in the strength of association between couples of flourishing components across countries, supporting the mounting evidence that flourishing is a psychological process which is rooted in the socio-cultural contexts of individuals.

The countries included in the GFS (see Methods for the full list) differ from each other in ways that are relevant to individual well-being^27^. For instance, financially, the GDP per capita of the included countries ranged from USD 80,412 in the United States (ranked in the top 25 globally) to USD 1,326 in Tanzania (ranked in the bottom 25 globally),12 out of 22 countries were classified as emerging and developing economies^28^. The proportion of the population reported as non-religious ranged from 73% in Sweden to 2% in Nigeria^29^. For national-level safety perception, the samples included countries ranked the 9^th^ (Japan) to 147^th^ (Turkey) according to Global Peace Index^30^. For social trust, the proportion of people who agreed that “most people can be trusted” ranged from 62.8% in Sweden to 4.6% in Indonesia^31^. These examples illustrate salient differences across countries that may shape how people conceptualize and pursue individual flourishing. Whereas our approach is grounded in a sociocultural perspective which posits that flourishing is deeply informed by culturally rooted values and meanings (e.g^18^.,), we refrain from proposing directional hypotheses about how these factors influence the structure of individual flourishing. Accordingly, we treat these contextual differences as theoretically relevant but adopt an exploratory stance, anticipating heterogeneity in the network structure of individual flourishing across countries.

Beyond validating the SFM among a diverse set of countries, our study addressed a substantial gap in the well-being literature about how components of flourishing interact with each other and whether those interactions are similar or different across countries. Results of this study can offer insights into the dynamics sustaining the individual flourishing, making an invaluable contribution in generating confirmatory hypothesis to be tested in future studies. Instead of viewing individual flourishing as one homogenous psychological phenomenon, our systems approach helps understand the different “symptom” (terminology borrowed from Keyes’ [2002] flourishing model) profiles of individual flourishing, such that the same concept of flourishing has diverse expressions in various geographical locations. Indeed, analyzing individual flourishing in large scale representative samples the countries included in the GFS can help evaluate how broadly the network structure of individual flourishing might apply to a large portion of the global human population, recognizing the vastly different ecological landscapes in which people around the world are embedded. More generally, leveraging the GFS data for a cross-cultural network analysis focused on individual flourishing can contribute to addressing the long-standing criticism that existing evidence about human flourishing tends to overrepresent the Western, educated, industrialized, rich, and democratic population^32,33^.

## Methods

### Study sample

Wave 1 of the GFS included nationally representative samples from Argentina, Australia, Brazil, Egypt, Germany, Hong Kong (Special Administrative Region of China), India, Indonesia, Israel, Japan, Kenya, Mexico, Nigeria, the Philippines, Poland, South Africa, Spain, Sweden, Tanzania, Türkiye, United Kingdom, and the United States. The countries were selected to (a) maximize coverage of the world’s population, (b) ensure geographic, cultural, and religious diversity, and (c) prioritize feasibility in line with existing data collection infrastructure. Data collection was conducted by Gallup Inc. Data for Wave 1 was collected mainly during 2023, although the exact dates varied by country^34,35^. The mean age of the sample was 45.83 years (range = 18–99; *SD* = 17.67), with slightly more females (52.7%) represented than males (47%), 0.2% who identified in none of the former categories, and 0.1% who preferred not to answer. Each country’s sample size ranged from *n* = 1,473 (Turkey) to *n* = 38,312 (USA), which are presented in Table 2 alongside the average weighted zero-order correlations of each variable with all others in each country. The GFS survey assesses aspects of individual flourishing, such as happiness, health, meaning, character, relationships, and financial stability^7^, alongside other sociodemographic, political, religious, personality, childhood, community, and health variables. Gallup translated the GFS survey into multiple languages following the TRAPD (translation, review, adjudication, pretesting, and documentation) model for cross-cultural survey research. Extensive details about the translation, cognitive interviewing, and piloting testing phases of the GFS can be found elsewhere^34,36,37^. To ensure samples were nationally representative, Wave 1 of the GFS used different sampling schemes across countries based on availability of existing panels and recruitment needs^35^, for further details, see^34^).

**Table 2:** Average weighted zero-order correlations of each flourishing item within country (*M*_*α*_).

Country	Sample size	H	LS	MH	PH	SP	W	PG	GU	C	SR	E	WS	*M*_*row*_
Argentina	6724	0.418	0.427	0.391	0.301	0.358	0.408	0.255	0.193	0.353	0.379	0.211	0.175	0.322
Australia	3844	0.558	0.562	0.546	0.371	0.481	0.529	0.355	0.311	0.502	0.492	0.379	0.390	0.456
Brazil	13,204	0.466	0.473	0.439	0.349	0.481	0.461	0.297	0.253	0.414	0.416	0.228	0.192	0.372
Egypt	4729	0.256	0.256	0.217	0.219	0.240	0.241	0.171	0.136	0.246	0.234	0.219	0.227	0.222
Germany	9506	0.431	0.437	0.379	0.297	0.332	0.386	0.222	0.200	0.348	0.354	0.293	0.291	0.331
Hong Kong	3012	0.730	0.721	0.713	0.692	0.674	0.709	0.696	0.612	0.686	0.686	0.583	0.547	0.671
India	12,765	0.311	0.331	0.321	0.302	0.268	0.318	0.270	0.204	0.274	0.260	0.155	0.126	0.262
Indonesia	6992	0.389	0.405	0.408	0.376	0.369	0.400	0.371	0.330	0.369	0.392	0.221	0.194	0.352
Israel	3669	0.503	0.440	0.471	0.432	0.459	0.504	0.388	0.251	0.465	0.458	0.403	0.391	0.430
Japan	20,543	0.654	0.666	0.652	0.540	0.600	0.648	0.612	0.251	0.465	0.458	0.493	0.520	0.547
Kenya	11,389	0.250	0.227	0.226	0.250	0.226	0.252	0.218	0.206	0.241	0.247	0.178	0.179	0.225
Mexico	5776	0.435	0.455	0.432	0.364	0.402	0.439	0.334	0.264	0.406	0.420	0.223	0.151	0.360
Nigeria	6827	0.284	0.281	0.283	0.293	0.280	0.300	0.278	0.277	0.283	0.289	0.161	0.151	0.263
Philippines	5292	0.346	0.366	0.370	0.331	0.352	0.341	0.341	0.344	0.357	0.364	0.188	0.213	0.326
Poland	10,389	0.505	0.502	0.463	0.403	0.422	0.480	0.403	0.186	0.475	0.466	0.356	0.379	0.420
South Africa	2651	0.319	0.310	0.244	0.252	0.276	0.303	0.272	0.260	0.283	0.286	0.183	0.184	0.264
Spain	6290	0.433	0.434	0.390	0.313	0.351	0.423	0.242	0.218	0.361	0.368	0.210	0.192	0.328
Tanzania	9075	0.303	0.269	0.249	0.295	0.274	0.322	0.260	0.224	0.277	0.268	0.256	0.257	0.271
Türkiye	1473	0.439	0.445	0.417	0.324	0.394	0.428	0.326	0.237	0.341	0.375	0.353	0.339	0.368
United Kingdom	5368	0.563	0.560	0.531	0.372	0.480	0.535	0.366	0.340	0.496	0.487	0.377	0.387	0.458
United States	38,312	0.565	0.561	0.531	0.376	0.481	0.542	0.372	0.340	0.511	0.495	0.368	0.363	0.459
Sweden	15,068	0.560	0.565	0.520	0.381	0.462	0.539	0.359	0.236	0.490	0.494	0.372	0.373	0.446
*M*_*column*_		0.442	0.441	0.418	0.356	0.394	0.432	0.337	0.267	0.393	0.395	0.291	0.283	

*Note.* H = happy; LS = life satisfaction; MH = mental health; PH = physical health; SP = sense of purpose; W = life worthwhile; PG = promote good; GU = give up happiness; C = content with relationships; SR = satisfying relationships; E = worry about expenses; WS = worry about safety. *M*_*row*_ = row mean, *M*_*column*_ = column mean

### Measures

The present study uses the 12-item Secure Flourishing Measure (SFM; see Table 1 for the scale items of each of the six subscales and its scoring method) that was proposed by VanderWeele^7^ and has subsequently been psychometrically evaluated^8^. Items were answered on an 11-point response scale [0–10]. Descriptive statistics for the individual flourishing scores across countries are given in VanderWeele et al.^38^.

### Data analysis

The analyses were performed using R version 4.4.2 in RStudio. More information about the dataset is available at www.cos.io/gfs and https://osf.io/3jtz8/. Our analyses were pre-registered at 10.17605/OSF.IO/SNK4Y.

### Preliminary analysis

Missing data on observed variables ranged between 0.1% and 1.7% across countries and were omitted from subsequent analysis. Sample weights were applied to the correlation matrix in each single country before conducting further analyses.

### Measurement model comparison

Complex psychological constructs, such as flourishing, often resist being captured by a single psychometric model (e.g^39^.,). Thus, exploring multiple theoretically plausible models is crucial to identifying the most appropriate psychometric structure to fit the data before conducting further analyses. For this reason, we used the R package *psychonetrics* (version 0.13) to identify the best measurement model to explain the variance–covariance structure among the 12 GFS items. Specifically, six models were tested: (a) a *first-order confirmatory factor analysis model* in which the covariance between items is explained by six interrelated latent variables representing the dimensions of individual flourishing (i.e., happiness & life satisfaction, mental & physical health, meaning & purpose, character & virtue, close social relationships, and financial & material stability); (b) a *bi-factor model* in which all items load on their specific latent variable and also on a common factor; (c) a *hierarchical g-factor model* in which a second-order global factor explains the variance–covariance structure among the six latent variables; (d) a *latent network model* in which items’ covariance is explained by latent variables while the covariance structure between latent variables is modelled as a network; (e) a *residual network model* in which items’ covariance is explained by a single latent variable (potentially representing a common method bias) while the covariance between residuals is modelled through a GGM, and (f) a *gaussian graphical model* which represents pairwise conditional associations between the twelve indicators.

The competing models were evaluated according to comparative fit indices (AIC and BIC, lower values indicating better model fit), incremental fit indices (CFI, TLI, RFI, NFI; good fit when higher than 0.90), and the RMSEA as an absolute fit index (good fit when lower than 0.08^40,41^;). If the 12-item network model shows a better fit compared to the others, this confirms the adequacy of the network model as the preferred measurement model accounting for potential common method bias.

### Psychometric network analysis

Psychometric Network Analysis represents psychological phenomena as systems of interacting elements^42^. The basic network structure is composed of nodes, represented by variables, and edges, represented by pairwise conditional associations between couple of nodes while conditioning on the remaining nodes in the network. When an edge between two variables is absent (i.e., two nodes are disconnected in the network), this means that the association between the two variables can be explained by other variables in the network (i.e., conditionally independent). We used Gaussian Graphical Models (GGM) for multivariate normal data that model conditional associations between variables as partial correlations. To estimate a global flourishing network, the 12 GFS items were included as the nodes of the network, and partial correlations were estimated between them. The valence (positive or negative) and the strength (edge weight) of edges can be inspected to describe the connection between flourishing constituents.

### Meta-analytic gaussian network aggregation (MAGNA)

To investigate differences in the network structure across countries, a Meta-analytic Gaussian Network Aggregation (MAGNA) model was applied using *psychonetrics* 0.13^43^, which is based on (full information) maximum likelihood estimation, following Isvoranu and colleagues’ (2021) procedure. All networks were visualized using *qgraph*^44^.

MAGNA is based on a multi-dataset approach to estimate a pooled GGM over multiple correlation matrices^43,45^. To inquire about the existence of substantial heterogeneity over and above the sampling variation we first compared two models. The first model is a Gaussian Graphical Model (GGM) in which the network structure of partial correlations is allowed to assume unique values in each country. The obtained *single country saturated model* assumes that each country has a unique configuration of flourishing constituents. Then, a multi-group saturated model (*pooled saturated model*) was estimated, in which all edge weights were constrained to be equal across countries. The two models were then compared in their fit by examining two information criteria, the AIC and the BIC, where lower values indicate a better fit of the model. If the *single country saturated model* shows the best fit to the data, this can be interpreted as the existence of some degree of heterogeneity across countries. In this case, it is possible to proceed to the estimation of a random-effects meta-analytic Gaussian network aggregation model (random-effects MAGNA) which takes into account the variance of the correlational structure across countries. In a random-effects MAGNA model, for each correlation (marginal pairwise correlation) between two flourishing components is estimated a mean (fixed-effects) and a variance–covariance structure (random-effects) reflecting the heterogeneity between studies and sampling variation^43,45^. The means of sample correlations can be modelled as a GGM to obtain a common cross-country network (pooled MAGNA network), while the variance–covariance matrix of random effects on the implied correlational structure allows to assess cross-country heterogeneity. Parameters estimated through maximum likelihood estimation and the *nlminb* optimization algorithm are as follows: a) edge weights (partial correlation coefficients) of the pooled MAGNA network to examine the strength of associations between couples of flourishing components after conditioning on all other components in the network; b) standard errors of these edge weights which can be used to obtain p-values and calculate 95% confidence intervals (CIs); and c) standard deviations of random effects to interpreted cross-country heterogeneity in the correlational structure.

### Pooled MAGNA centrality indices

We examined two centrality measures for the pooled MAGNA network and for each single-country networks: expected influence^46^ and predictability (*R*^2^^47^,). Expected influence of a node is obtained by summing all the edge weights taking into account their sign (positive or negative), so that a high number of positive and strong connections will result in a higher expected influence value. Predictability, on the other side, quantifies how much variance of a node can be explained by the other nodes in the network. Centrality indices were estimated with *networktools*^48^.

To evaluate the stability of the obtained centrality indices from the pooled network, we used parametric bootstrapping following the approach by^45^. This approach employs sampling techniques to estimate significant differences in centrality between two nodes in a network. We generated 1,000,000 network models based on the estimated parameter variance–covariance matrix of the pooled MAGNA network edge weights. For each variable pair and centrality index, we calculated the proportion of times the difference fell below zero, as well as those where it exceeded zero. Finally, we took the lowest of these proportions and multiplied it by two to obtain a *p*-value for a two-sided difference test. This enables us to reject the null hypothesis of equal centrality at various significance levels using these p-values. Because this approach involves multiple comparisons of the centrality indices of all twelve nodes with one another, we applied a Bonferroni corrected level of 0.0008.

## Results

### Measurement model comparison

**Fig. 1:**
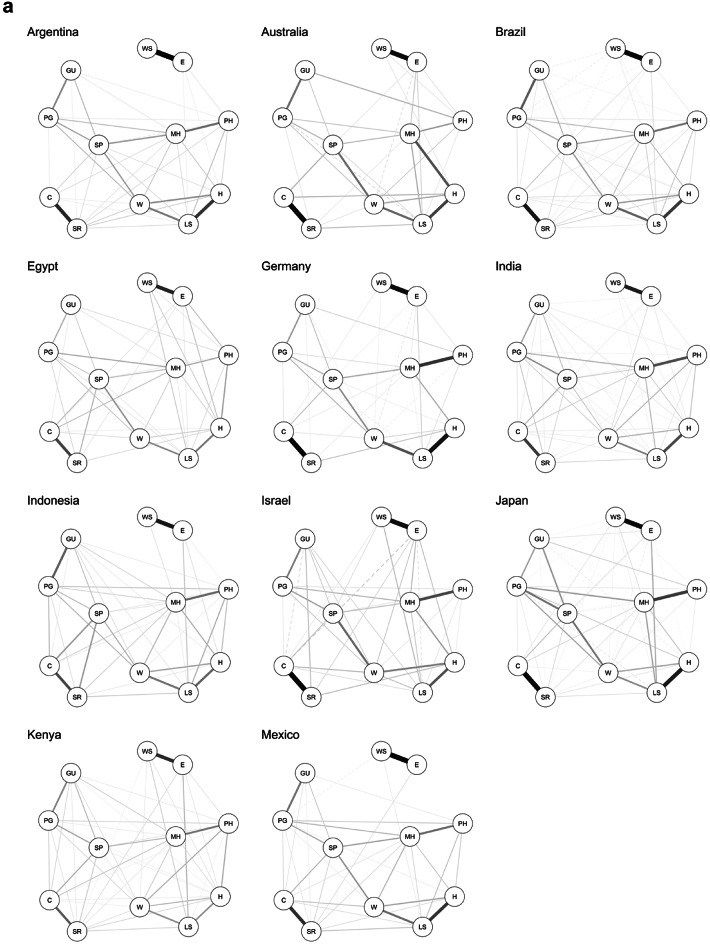

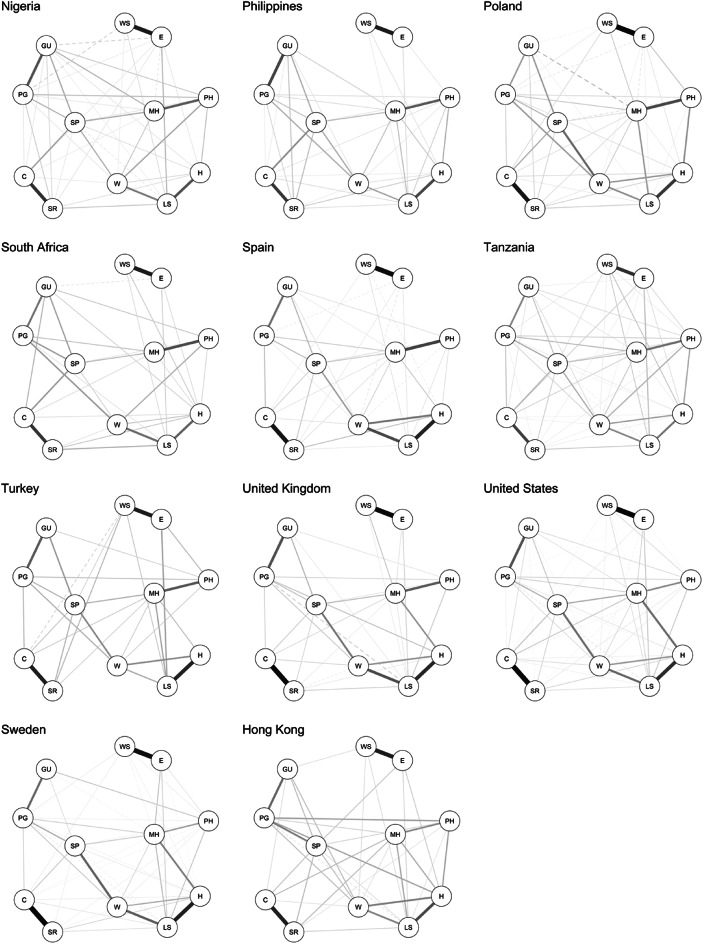
(**a**) Single-country flourishing networks of the first 11 countries (in alphabetical order) of the 22 countries. Note. Edge thickness indicates edge weight, solid edges indicate positive partial correlations, dashed edges indicate negative partial correlations. H = happiness; LS = life satisfaction; MH = mental health; PH = physical health; SP = sense of purpose; W = life worthwhile; PG = promote good; GU = give up happiness; C = content with relationships; SR = satisfying relationships; E = worry about expenses; WS = worry about safety. (**b**) Single-country flourishing networks of the last 11 counties (in alphabetical order) of the 22 countries. Note. Edge thickness indicates edge weight, solid edges indicate positive partial correlations, dashed edges indicate negative partial correlations. H = happiness; LS = life satisfaction; MH = mental health; PH = physical health; SP = sense of purpose; W = life worthwhile; PG = promote good; GU = give up happiness; C = content with relationships; SR = satisfying relationships; E = worry about expenses; WS = worry about safety.

Among the tested measurement models, the gaussian graphical network model (GGM) was the best to represent the data generating mechanism (Table 3). A Meta-analytic Gaussian Network Aggregation (MAGNA) model was estimated to identify a common-country flourishing network and examine the cross-country differences in the pairwise associations between flourishing components. The *single country saturated model* in which each country was allowed to express a different path of associations between flourishing components performed better according to both AIC (5,479,091.52) and BIC (5,493,891.46) than the *multigroup saturated model* (AIC = 5,624,983.91; BIC = 5,625,656.63), in which the network structure was constrained to be identical across the 22 countries. These results indicated the presence of substantial cross-country heterogeneity within the flourishing network after accounting for sample variations that could be further explored by testing a random-effects MAGNA model. Figures 1a-b present the single country networks extracted from the best fitting model in which only significant edges were retained by means of thresholding (alpha 0.05). Corresponding numeric values of edge weights can be consulted in Table S1 in supplementary materials.

**Table 3:** Model fit indices for preliminary analysis.

Model	*df*	*χ*^*2*^	*χ*^*2*^_*difference to network model (p-value)*_	*NFI*	*TLI*	*RFI*	*CFI*	*AIC*	*BIC*	*RMSEA*
First-order CFA model	39	21,058.84	21,049.82 (< .001)	.98	.97	.97	.98	10,112,874.14	10,113,393.97	.05 [.05, .05]
Bi-factor model	42	35,153.39	35,144.37 (< .001)	.97	.95	.95	.97	10,126,962.69	10,127,451.94	.06 [.06, .07]
g-factor/second-order model	48	35,343.95	35,334.93 (< .001)	.97	.96	.96	.97	10,127,141.26	10,127,569.35	.06 [.06, .06]
Latent network model	39	21,058.35	21,049.33 (< .001)	.98	.97	.97	.98	10,112,873.65	10,113,393.48	.05 [.05, .05]
Residual network model*	12	25.05	16.03 (.025)	1.0	1.0	1.0	1.0	10,091,894.35	10,092,689.39	.00 [.00, .00]
Network model	5	9.02		1.0	1.0	1.0	1.0	10,091,892.32	10,092,758.71	.00 [.00, .00]

*Note*. *convergence issues.

### Meta-analytic gaussian network aggregation (MAGNA)

**Fig. 2:**
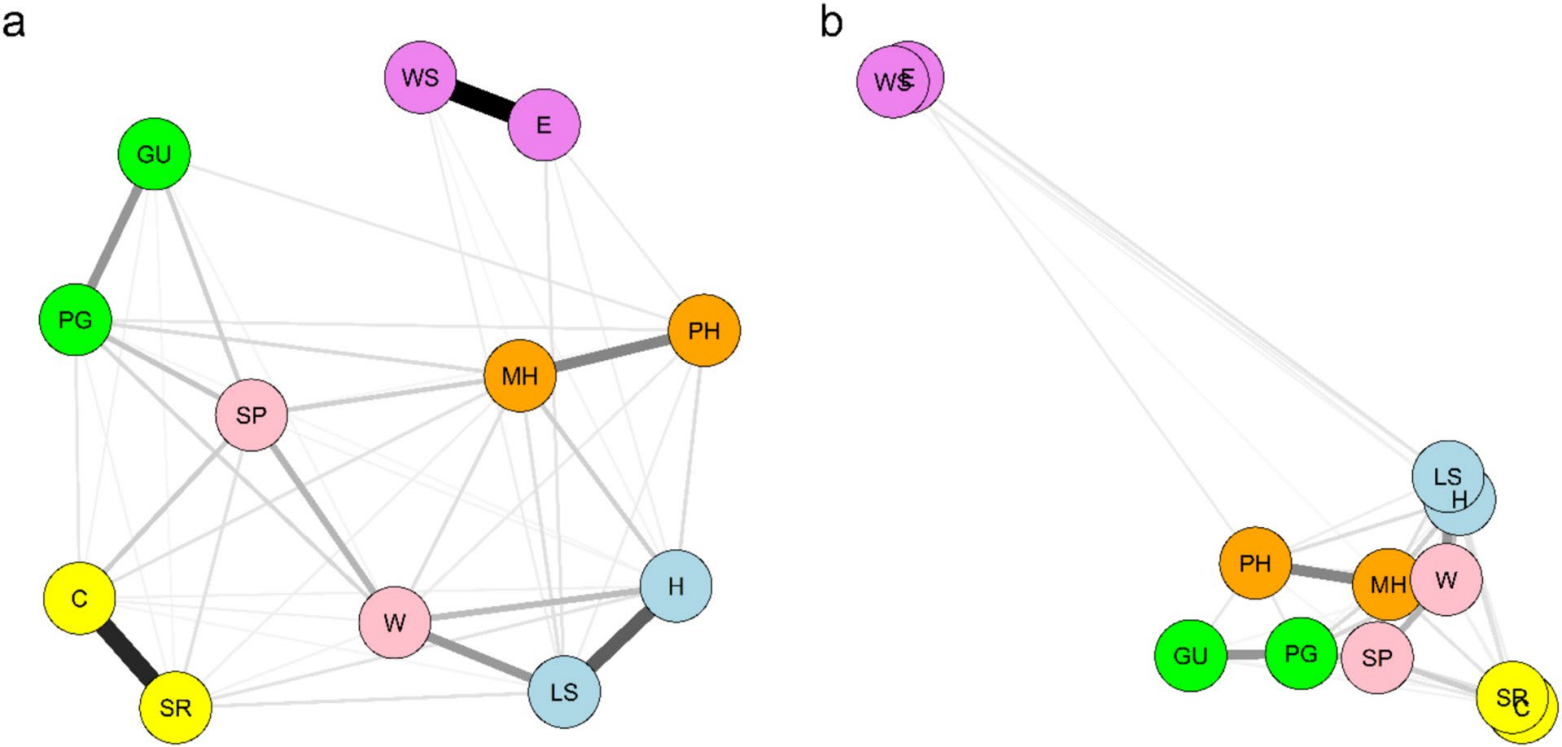
Common-country individual flourishing network generated from the pooled Meta-Analytic Gaussian Network Aggregation (MAGNA). *Note.* (**a**) Average layout. (**b**) Principal Component Analysis layout. Nodes represent flourishing components, and edges represent partial correlation coefficients (edge thickness indicates edge weight). Nodes with the same color belong to the same dimension. H = happiness; LS = life satisfaction; MH = mental health; PH = physical health; SP = sense of purpose; W = life worthwhile; PG = promote good; GU = give up happiness; C = content with relationships; SR = satisfying relationships; E = worry about expenses; WS = worry about safety.

**Fig. 3:**
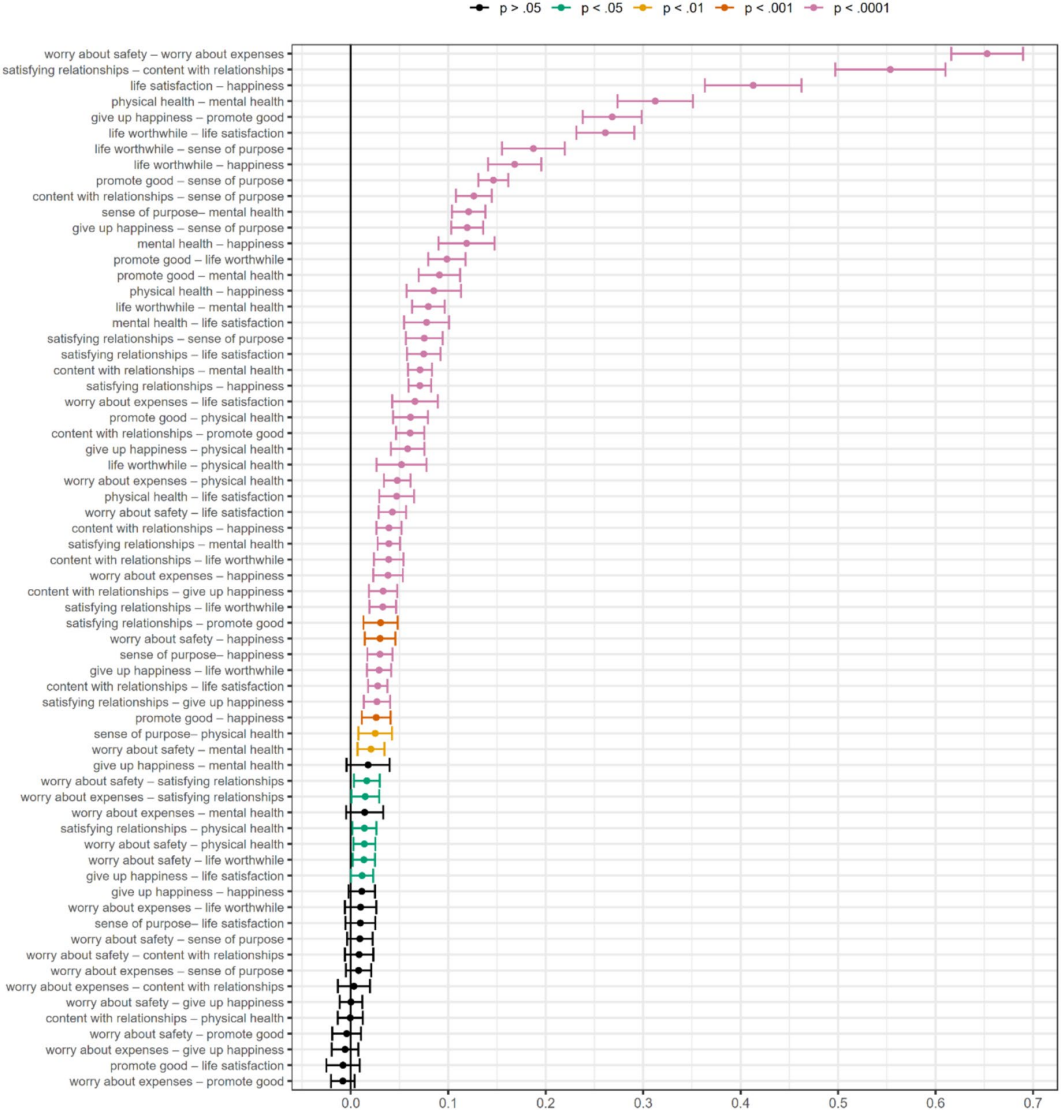
Estimated edge weights in the pooled Meta-Analytic Gaussian Network Aggregation (MAGNA) and 95% confidence regions based on their standard errors.

Given the best fitting model indicating that the individual flourishing network varied across countries, we estimated a random-effects MAGNA to derive a common cross-country network and examined the most discrepant associations between the individual flourishing components. Figure 2 shows the estimated pooled MAGNA for the components using the Fruchterman-Reingold algorithm and principal component analysis (PCA) layout. Figure 3 presents the 95% confidence intervals (CIs) for the estimated edge weights in the pooled MAGNA network. A total 51 edges out of 66 possible partial correlations were significantly different from zero and positive. As expected, the strongest partial correlations across countries were between items in the same individual flourishing domain, with edges ranging from 0.65 (financial & material stability) to 0.19 (meaning & purpose). However, several consistent partial correlations connected items from distinct domains, such as *life satisfaction* with *life worthwhile* (0.26), *sense of purpose* with items such as *promote good* (0.15), *content with relationships* (0.13), *give up happiness* (0.12), and *mental health* (0.12), as well as *happiness* with *life worthwhile* (0.17) and *mental health* with (0.12). The visualization in Fig. 2 based on the PCA layout shows that the two financial & material stability components are strongly associated with each other but less strongly associated with other individual flourishing components, as the strongest association is the one between *worry about expenses* and *life satisfaction* (0.07).

### Centrality indices and cross-country heterogeneity

**Fig. 4:**
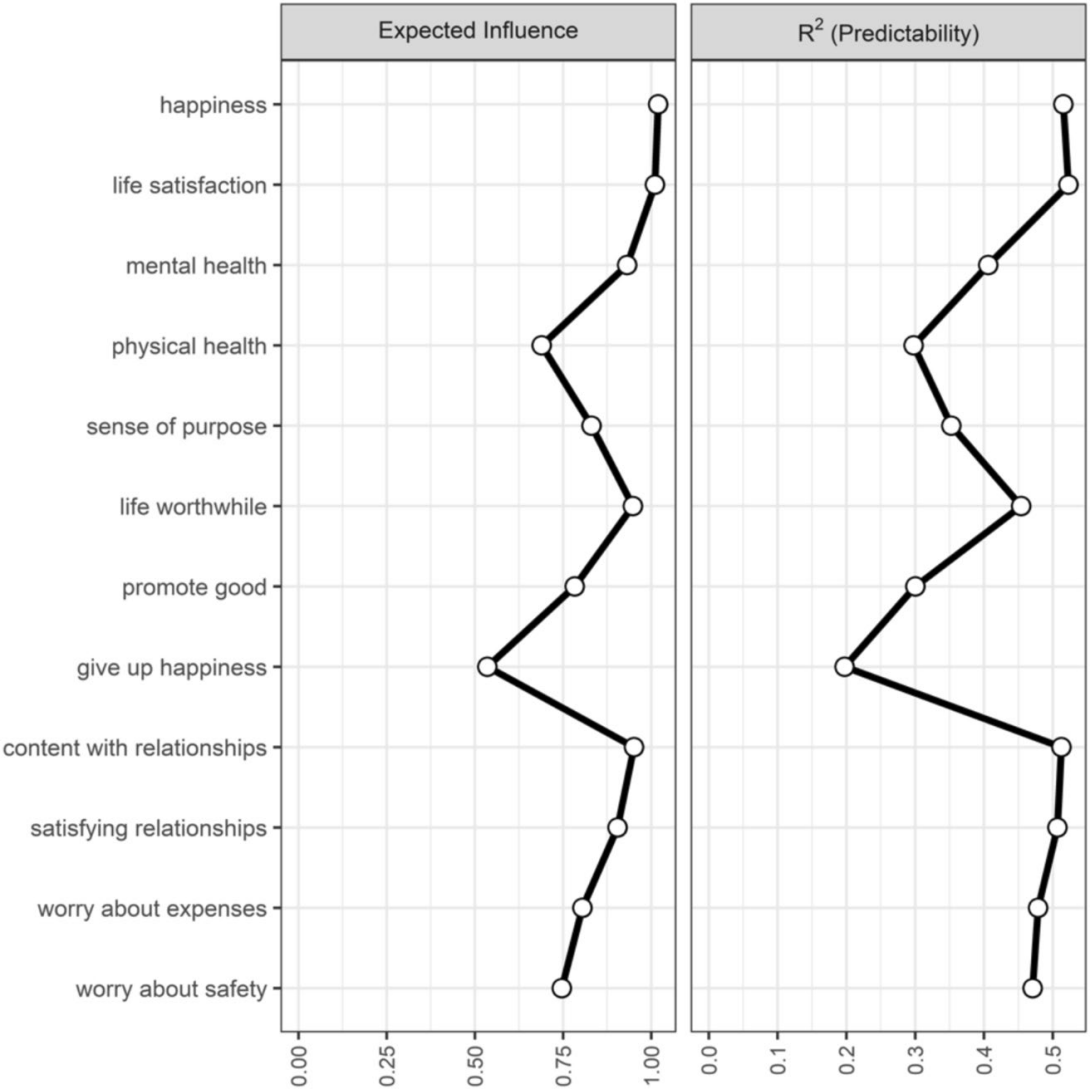
Centrality Indices of the pooled Meta-Analytic Gaussian Network Aggregation (MAGNA).

**Fig. 5:**
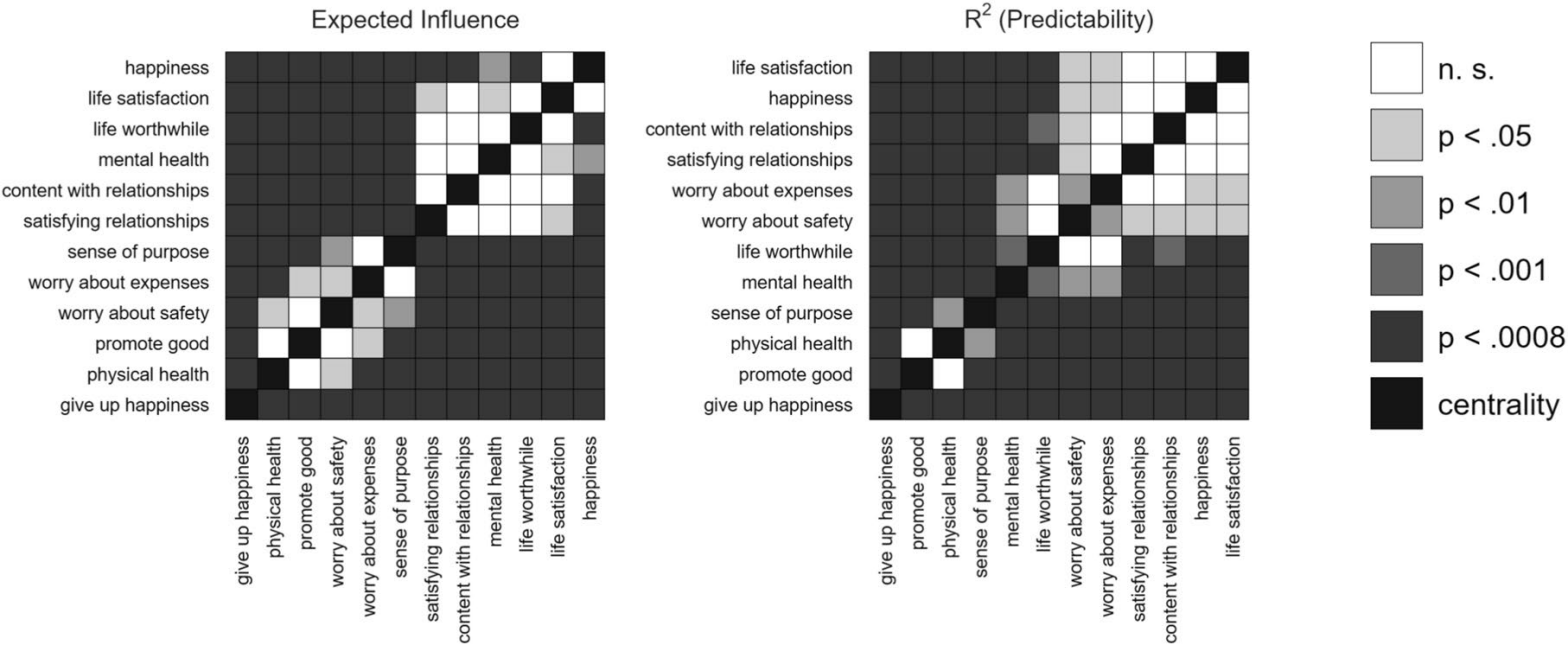
Centrality difference plots obtained through a parametric bootstrap. Each block indicates the significance of the difference between centrality indices of two nodes. These were obtained by sampling 1,000,000 network structures from the estimated asymptotic parameter variance–covariance matrices. The α = .0008 level corresponds to a Bonferroni corrected α-level of .05 rounded to 4 digits based on comparing the centralities of all 12 nodes with each other.

Centrality indices of nodes of the pooled MAGNA are reported in Table 4 and represented in Fig. 4. Overall, *happiness* (1.02) and *life satisfaction* (1.01) showed a significantly higher expected influence compared to the other nodes, followed by a set of nodes including *content with relationships* (0.95), *life worthwhile* (0.95), *mental health* (0.93) and *satisfying relationships* (0.91). *Life satisfaction* (0.52), *happiness* (0.51), *content with relationships* (0.51) and *satisfying relationships* (0.51) were also the ones with a significantly higher predictability, confirming their central role in the individual network of flourishing. Conversely, *give up happiness*, followed by *promote good* and *physical health*, were the least connected node within the network, showing significantly lower expected influence (0.53—0.78) and predictability (0.20—0.30) compared to the other nodes. The average expected influence and predictability (R2) were 0.85 and 0.42. See Supplemental Table S2-S3 for the centrality measures for single countries. Figure 5 shows the results of the parametric bootstrapped difference tests, which demonstrated substantial stability of the obtained centrality indices from the pooled network.

**Table 4:** Summary of centrality indices of the Pooled MAGNA.

Variable	*Expected Influence*	*R*^*2*^* (Predictability)*
Life satisfaction	1.010	0.523
Mental health	0.931	0.406
Happiness	1.019	0.515
Satisfying relationships	0.904	0.507
Content with relationships	0.951	0.512
Life worthwhile	0.948	0.454
Promote good	0.783	0.300
Sense of purpose	0.831	0.352
Worry about safety	0.746	0.471
Worry about expenses	0.805	0.478
Physical health	0.689	0.298
Give up happiness	0.535	0.197

**Fig. 6:**
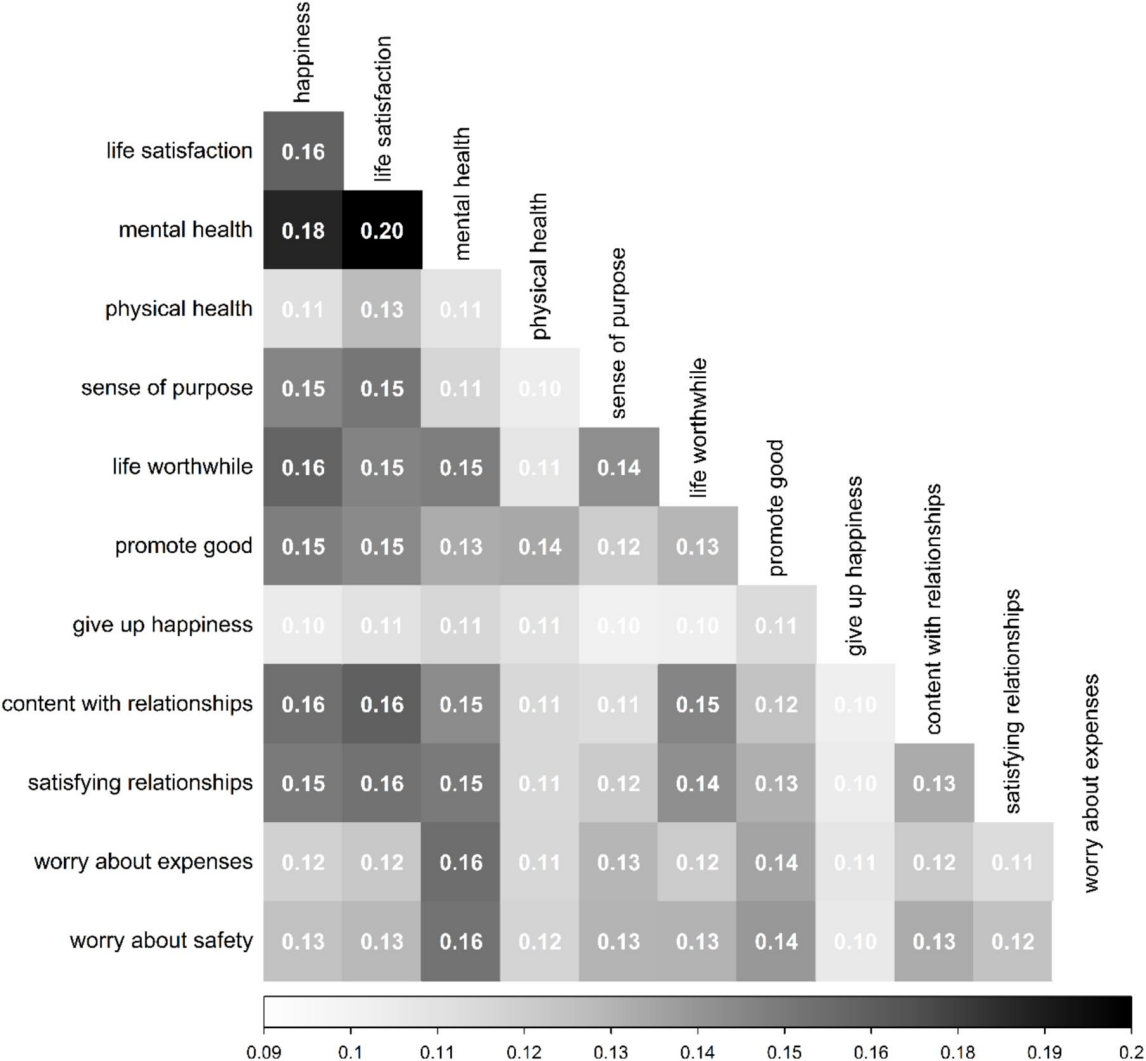
Estimated random effect standard deviations of unconditional associations between the flourishing components. Higher values indicate larger differences between countries in correlational structure.

To examine which edges differed the most across countries in the effect size or the direction of the association, we inspected the random effect standard deviations for the correlational structure implied by the pooled MAGNA network. The average standard deviation was 0.12, and estimates ranged from 0.08 to 0.19. As shown in Fig. 6, the edges that differed the most between the countries were *mental health* with *life satisfaction* (0.19) and *mental health* with *happiness* (0.18), followed by a vast number of edges with random effects ranging between 0.16 and 0.14. The edge that was most similar across countries was *worry about safety* with *worry about expenses* (0.08), followed by all the edges involving *give up happiness* (with estimates ranging from 0.09 to 0.11). Some insights can be gathered by considering the differences in edge weights between countries presented in Figures 1a-b and Supplemental Table S1. For example, the most discrepant edge, which was between *life satisfaction* and *mental health*, was positive for most of the countries (ranging from 0.03 in Nigeria to 0.18 in Poland) except for Kenya, South Africa, in which these two nodes were not significantly different from zero, denoting a case of conditional independence. For Tanzania, the components *life satisfaction* and *mental health* even showed a marginal negative connection (-0.03). The second most discrepant connection, which was between *mental health* and *happiness*, showed positive interconnections ranging from 0.03 (Tanzania) to 0.36 (Australia) in all countries except for Nigeria in which the conditional association was zero.

## Discussion

Our study purpose was to examine the psychometric network of individual flourishing across 22 countries with cross-sectional data from the first wave of the GFS. We found from the pooled network analysis that most edges between 12 SFM items^7^ shared positive partial correlations in both the pooled and the single-country networks. Finding all significant edges among constituents of individual flourishing network being positive is consistent with the theoretical postulations that these twelve components are basic constituents of the process of individual flourishing across cultures^7,16^. Only few partial correlations showed marginal negative values in single-country networks, and none of these edges was negative at a statistically significant level (*p* < 0.05). This suggests that the global findings reveal these as overall negligible (i.e., close to zero) edges rather than signaling negative ones in specific locations.

Consistent with the theoretical structure of the Secure Flourish Measure, edges between couples of components belonging to the same cluster (e.g., financial & material stability) were generally stronger than those between components from different domains. However, we noted consistent partial correlations between components belonging to different dimensions. These conditional dependencies between components in different domains underscore the importance of including them in the individual flourishing network and investigating the role of each to the network structure. Examples of positive edges that were consistent across all countries (excluding those within the same domain) were for the *life worthwhile* component with both the *happiness* and *life satisfaction* components, as well as for the *sense of purpose* component with *content with relationships*, *promote good*, and *give up happiness* components, suggesting the robustness of these positive links across the constituents of individual flourishing.

Absent edges (those which are not statistically different from zero) can also inform the functioning of the individual flourishing network. Some flourishing components were not connected in the pooled network (i.e., conditional independence), meaning that they were not uniquely associated after considering their network of associations with other components. Instances of conditional independence between specific nodes do not exclude the possibility that two nodes are connected indirectly through shared linkages with at least one other variable in the network. For instance, consistent with results reported in Höltge et al.^16^, the two items of the character and virtue dimension (*promote good* and *give up happiness*) were not directly related to *life satisfaction*, and only *promote good* was slightly associated with *happiness* in the pooled network, even though the zero-order correlations between these components were between 0.26 and 0.36. One potential explanation for such findings could be that the dimension of life satisfaction and happiness is connected with character and virtue via other flourishing components, for example the items belonging to the meaning and purpose dimension that, as can be visually seen from the network representation, appear to act as a bridge between the two (see also^49^ and Chen et al., 2021 for a discussion on the *indirect* effects on life satisfaction through other individual flourishing constituents).

Another interesting finding in the pooled network is that the edge between *life satisfaction* and *life purpose* is absent, despite showing a zero-order correlation of 0.41 (Table 5), meaning that these two nodes are conditionally independent after conditioning on all the others. However, *life worthwhile* is positively associated in the network with both *life satisfaction* and *life purpose*, acting as a "bridge” between the two components. This pattern reveals an interesting flourishing dynamic, suggesting that being able to understand personal life goals is related to life satisfaction only when people’s intentions are oriented toward worthwhile actions. This pattern was found in most individual countries (see supplementary materials), confirming the generalizability of this dynamic. However, because our data were cross-sectional and did not control for possible confounds, these results should not be interpreted causally.

**Table 5:** Edge weights of the pooled MAGNA (upper diagonal) and overall weighted correlation matrix (lower diagonal) for the total sample.

Variable	H	LS	MH	PH	SP	W	PG	GU	C	SR	E	WS
H	1.000	**0.410**	**0.120**	**0.085**	**0.030**	**0.170**	**0.026**	0.011	**0.039**	**0.071**	**0.038**	**0.030**
LS	0.657	1.000	**0.078**	**0.047**	0.001	**0.260**	-0.008	**0.012**	**0.028**	**0.075**	**0.066**	**0.043**
MH	0.513	0.466	1.000	**0.310**	**0.120**	**0.080**	**0.091**	0.018	**0.071**	**0.039**	0.001	0.021
PH	0.437	0.387	0.561	1.000	**0.025**	**0.052**	**0.061**	**0.058**	0.000	**0.014**	**0.048**	**0.014**
SP	0.433	0.407	0.519	0.393	1.000	**0.190**	**0.150**	**0.120**	**0.130**	**0.075**	0.008	0.009
W	0.580	0.603	0.515	0.426	0.523	1.000	**0.099**	**0.029**	**0.039**	**0.033**	0.010	**0.013**
PG	0.363	0.339	0.432	0.358	0.477	0.430	1.000	**0.270**	**0.061**	**0.031**	-0.008	-0.004
GU	0.289	0.259	0.329	0.300	0.395	0.334	0.464	1.000	**0.033**	**0.027**	-0.006	0.000
C	0.456	0.443	0.482	0.359	0.517	0.474	0.418	0.336	1.000	**0.550**	0.003	0.009
SR	0.462	0.461	0.465	0.357	0.494	0.467	0.390	0.309	0.731	1.000	**0.015**	**0.016**
E	0.300	0.326	0.232	0.210	0.162	0.254	0.110	0.086	0.196	0.206	1.000	**0.650**
WS	0.278	0.301	0.210	0.185	0.140	0.237	0.097	0.069	0.181	0.189	0.734	1.000

*Note.* Significant edge weights (p < 0.05) are reported in **bold**. H = happy; LS = life satisfaction; MH = mental health; PH = physical health; SP = sense of purpose; W = life worthwhile; PG = promote good; GU = give up happiness; C = content with relationships; SR = satisfying relationships; E = worry about expenses; WS = worry about safety.

One last consideration is dedicated to financial and material stability. In our study, the two related items are strongly associated together but show only few small associations with other flourishing components (e.g*., life satisfaction* and *happiness*), while in Holtge et al. (2023) the global indicator of financial & material stability was not related to any other individual flourishing domains. However, it is difficult to directly compare the two studies given their many differences, including the age range, coverage of cultures, and unit of analysis (i.e., psychometric networks estimated at the domain vs. item level).

While some similarities were found in the individual flourishing networks across the countries, considerable cross-country heterogeneity was also demonstrated by substantial random effects of the edges and differences in the overall connectivity of the individual flourishing network across countries. For example, among the positive edges, the most discrepant ones were *mental health–life satisfaction* and *mental health–happiness.* In some countries, such as Australia, Sweden or Hong Kong, *mental health* showed a positive unique association with both *life satisfaction* and *happiness*. In other countries, such as Spain or South Africa, *mental health* was only associated with *happiness*, whereas in Nigeria *mental health* was uniquely associated with *life satisfaction* but not with *happiness*. There may be several potential reasons for the cross-country heterogeneity that was observed, such as country differences in population demographics (e.g., some countries have older populations than others) that may be related to individual flourishing (see^38^), cross-country differences in sociocultural factors that could play a salient role in shaping the interconnectivity between individual flourishing components (e.g., individualism vs collectivism), as well as methodological factors that pose challenges to multinational research (e.g., seasonality effects, cultural differences in how people interpret and respond to items). Even though the investigation of predictors of flourishing functioning goes beyond the scope of the present work, which has the primary aim of describing the main differences in the individual network of flourishing across countries within a cross-sectional framework, these results should be taken as the starting point to formulate confirmatory hypothesis to be tested in future studies.

### Strengths and limitations

Humanity shares many flourishing and prosperity goals in common, partially reflected in, for example, the United Nations’ Sustainable Development Goals (SDGs). Alongside the consensus among countries to strive for these SDGs, the evidence of heterogeneity of the individual flourishing network across the countries suggests that policies and interventions to promote human flourishing need to consider contextual, country-specific dynamics. The systems approach may also help identify “leverage points”, or the nodes with the most impact on other ones, that can be leveraged in service of a global agenda oriented toward the promotion of human flourishing. This has both important theoretical and practical implications for future research focusing on the “entry points” of interventions and how to “destabilize” the low-flourishing network. However, we caution against a one-size-fits all approach to policies and interventions to promote human flourishing in different cultural contexts. For decades, scientists have designed and tested many interventions to promote happiness and well-being with various degrees of success (e.g.,^50^). Attempts to replicate effective strategies in different countries will require careful examination of the similarities and differences between their individual flourishing networks and be sensitive to the unique sociocultural particularities that shape challenges and opportunities for human flourishing in each context.

The GFS translated and administered the same flourishing questions across 22 countries. However, one challenge with multinational survey data is to obtain evidence of sufficient construct validity to sustain the applicability and interpretability of the items across countries. The cross-cultural adaptation of the SFM followed a rigorous translation process. Moreover, Cowden et al.^36^ conducted cognitive interviews from 116 individuals from each GFS country to explore similarities and differences in comprehension of some GFS survey items (including the *life worthwhile* and *give up happiness* items from the SFM). Although they found substantial evidence in support of an overarching cross-country theme that suggested item comprehension was comparable across the countries, there was also some cross-country variability. Thus, the interpretation of observed network differences should take into consideration meaningful cultural variation in individual flourishing as well as potential differences in the way that people from different cultural contexts might understand, reflect on, and respond to the SFM items.

Although samples were weighted to be roughly nationally representative of the populations from which they were drawn^34,35^, it is possible that some groups (e.g., those at the lowest end of the health spectrum) may be underrepresented in the sample due to nonresponse, inaccessibility, or sampling frame limitations^51^. Readers should be mindful of the potential bias in network structures across countries.

Another limitation stems from the fact that our findings are based on a brief multidimensional measure that focuses strictly on components of individual flourishing that are nearly universally desired. Although this briefer measure is often preferable in large-scale surveys like the GFS because of the limited number of items that could be administered, the resultant individual flourishing networks might not include constituents that may also be salient within a particular context. Future research might consider applying a bottom-up, etic approach to identify other constituents of individual flourishing that could deepen our understanding of human flourishing in various cultures.

Importantly, the dynamic nature of a psychometric network suggests that the patterns identified at one time point may change and function differently when the system is transitioning into a new state. For example, people with a high variability in flourishing are likely to transition into a lower or higher state of flourishing than before (i.e., critical fluctuation). Our cross-sectional data limited our ability to investigate how the constituents of individual flourishing influenced each other and the plasticity of the individual flourishing network. Longitudinal studies, together with the consideration of micro- (e.g., age, gender) and macro-level covariates (e.g., cultural values), are necessary to further examine directionality and reciprocity of the individual flourishing components, as well as to unpack the identified homogeneity and heterogeneity across countries.

## Conclusion

In conclusion, consistent with previous smaller-scale research, our study on the flourishing network with over 200,000 participants across 22 countries reveals that all components are interconnected and interdependent, and a single global network of individual flourishing falls short of capturing the heterogeneity of these interconnectedness across countries. Beyond the insights provided by Höltge et al.^16^, our study contributes by identifying distinct profiles of individual flourishing in different contexts. Such understanding is urgently needed by the flourishing literature to establish baseline profiles across various geographical regions with nationally representative samples. Our findings help shed further light on potential entry points for policy and intervention, which aggregated domain-level analyses may overlook. Considering the unique individual flourishing networks that were observed for each country, our findings will hopefully ignite further discussion on and development of context-specific and culturally sensitive ways to promote greater good for all.

## Fundings

The GFS was supported by funding from the John Templeton Foundation (grant #61,665), Templeton Religion Trust (#1308), Templeton World Charity Foundation (#0605), Well-Being for Planet Earth Foundation, Fetzer Institute (#4354), Well Being Trust, Paul L. Foster Family Foundation, and the David and Carol Myers Foundation. The opinions expressed in this publication are those of the authors and do not necessarily reflect the views of these organizations.

## Supplementary Information

Supplementary Information.

## Data Availability

Data for Wave 1 of the Global Flourishing Study is available through the Center for Open Science upon submission of a pre-registration, and will be openly available without pre-registration beginning Spring 2026. Subsequent waves of the Global Flourishing Study will similarly be made available. Please see https://www.cos.io/gfs-access-data for more information about data access. All data generated or analysed during this study are included in this published article. Analysis codes are shared at on the Open Science Framework at this link: https://osf.io/6yafb/.

## References

[1] VanderWeele, T. J. et al. Flourishing in critical dialogue. *SSM - Mental Health* **3**, 100172. 10.1016/j.ssmmh.2022.100172 (2023).

[2] Organisation for economic co-operation and development. *Beyond GDP: Measuring what counts for economic and social performance.*10.1787/9789264307292-en (2018).

[3] Organisation for economic co-operation and development. *Measuring subjective well-being across OECD countries*10.1787/f5199579-en. (2024).

[4] Keyes, C. L. Promoting and protecting mental health as flourishing: a complementary strategy for improving national mental health. *Am. Psychol.* **62**(2), 95–108. 10.1037/0003-066x.62.2.95 (2007). 17324035 10.1037/0003-066X.62.2.95

[5] Seligman, M. E. P. *Flourish: A visionary new understanding of happiness and well-being* (Free Press, 2011).

[6] VanderWeele, T. J. & Lomas, T. Terminology and the well-being literature. *Affective Science* **4**(1), 36–40. 10.1007/s42761-022-00153-2 (2023). 37070016 10.1007/s42761-022-00153-2 PMC10104989

[7] VanderWeele, T. J. On the promotion of human flourishing. *Proc. of the National Academy of Sciences, 114*(31), 8148-8156. 10.1073/pnas.1702996114 (2017) 10.1073/pnas.1702996114 PMC5547610 28705870

[8] Weziak-Bialowolska, D., McNeely, E. & VanderWeele, T. J. Flourish index and secure flourish index–validation in workplace settings. *Cogent Psychol.* **6**(1), 1598926. 10.1080/23311908.2019.1598926 (2019).

[9] Lee, M. T. et al. Self-assessed importance of domains of flourishing: Demographics and correlations with well-being. *J. Posit. Psychol.* **16**(1), 137–144. 10.1080/17439760.2020.1716050 (2021).

[10] Weziak-Bialowolska, D. et al. Associations between the importance of well-being domains and the subsequent experience of well-being. *Sustainability* **15**(1), 594 (2022).

[11] Chen, Y., Cowden, R. G., Fulks, J., Plake, J. F. & VanderWeele, T. J. National data on age gradients in well-being among US adults. *JAMA Psychiat.* **79**(10), 1046–1047. 10.1001/jamapsychiatry.2022.2473 (2022). 10.1001/jamapsychiatry.2022.2473 PMC9403847 36001311

[12] Chen, Y. et al. Longitudinal associations between domains of flourishing. *Sci. Rep.* **12**(1), 2740. 10.1038/s41598-022-06626-5 (2022). 35177714 10.1038/s41598-022-06626-5 PMC8854559

[13] Cowden, R. G., Nakamura, J. S. & de la Rosa Fernández Pacheco P. A., Chen Y., Fulks J., Plake J. F., & VanderWeele T. J.,. The road to postpandemic recovery in the USA: A repeated cross-sectional survey of multidimensional well-being over two years. *Public Health* **217**(212), 217. 10.1016/j.puhe.2023.02.006 (2023). 10.1016/j.puhe.2023.02.006 PMC10010931 36924673

[14] Chen, Y. et al. Longitudinal associations between domains of flourishing. *Sci. Rep.* **12**(1), 2740 (2022). 35177714 10.1038/s41598-022-06626-5 PMC8854559

[15] Bialowolski, P. et al. Differences in multi-dimensional well-being among factory workers: Evidence from six countries. *Appl. Res. Quality Life* **18**, 2159–2180. 10.1007/s11482-023-10181-0 (2023). 10.1007/s11482-023-10181-0 PMC10209924 37359225

[16] Höltge, J. et al. A systems perspective on human flourishing: Exploring cross-country similarities and differences of a multisystemic flourishing network. *J. Posit. Psychol.* **18**(5), 695–710. 10.1080/17439760.2022.209378 (2023).

[17] Węziak-Białowolska, D., McNeely, E. & VanderWeele, T. J. Human flourishing in cross cultural settings: Evidence from the US, China, Sri Lanka Cambodia and Mexico. *Front. Psychol.* **10**, 1269. 10.3389/fpsyg.2019.01269 (2019). 31191421 10.3389/fpsyg.2019.01269 PMC6549439

[18] Willen, S. S., Williamson, A. F., Walsh, C. C., Hyman, M. & Tootle, W. Rethinking flourishing: Critical insights and qualitative perspectives from the US Midwest. *SSM-Mental Health* **2**, 100057. 10.1016/j.ssmmh.2021.100057 (2022). 34961852 10.1016/j.ssmmh.2021.100057 PMC8694651

[19] Willen, S. S. Flourishing and health in critical perspective: An invitation to interdisciplinary dialogue. *SSM Mental Health* **2**, 100045. 10.1016/j.ssmmh.2021.100045 (2022).

[20] Bronfenbrenner, U. *The ecology of human development: Experiments by nature and design* (Harvard University Press, 1979).

[21] Hakulinen, C. et al. Network structure of depression symptomology in participants with and without depressive disorder: The population-based health 2000–2011 study. *Soc. Psychiatry Psychiatr. Epidemiol.* **55**(10), 1273–1282. 10.1007/s00127-020-01843-7 (2020). 32047972 10.1007/s00127-020-01843-7 PMC7544719

[22] Heshmati, S., Oravecz, Z., Brick, T. R. & Roeser, R. W. Assessing psychological well-being in early adulthood: Empirical evidence for the structure of daily well-being via network analysis. *Appl. Dev. Sci.* **26**(2), 207–225. 10.1080/10888691.2020.1766356 (2022).

[23] Kossakowski, J. J. et al. The application of a network approach to health-related quality of life (HRQoL): Introducing a new method for assessing HRQoL in healthy adults and cancer patients. *Qual. Life Res.* **25**(4), 781–792. 10.1007/s11136-015-1127-z (2017). 10.1007/s11136-015-1127-z PMC4830856 26370099

[24] Shukla, M. et al. A network analysis of adolescent mental well-being during the coronavirus pandemic: Evidence for cross-cultural differences in central features. *Personality Individ. Differ.*10.1016/j.paid.2021.111316 (2022). 10.1016/j.paid.2021.111316 PMC8492750 34629577

[25] Stochl, J. et al. Identifying key targets for interventions to improve psychological wellbeing: Replicable results from four UK cohorts. *Psychol. Med.* **49**(14), 2389–2396. 10.1017/S0033291718003288 (2019). 30430959 10.1017/S0033291718003288 PMC6763534

[26] Crabtree S., English C., Johnson B. R., Ritter Z., & VanderWeele T. J. (2024). *Global flourishing study: 2024 questionnaire development report*. Gallup. https://osf.io/y3t6m 10.1186/s44263-025-00139-9 PMC12042518 40307930

[27] Case, B. et al. Beyond a single story: The heterogeneity of human flourishing in 22 countries. *Int. J. Wellbeing*10.5502/ijw.v13i4.3555 (2023).

[28] International Monetary Fund (2023). *World economic outlook database: Groups and aggregates information.* https://www.imf.org/external/datamapper/NGDPDPC@WEO/OEMDC/ADVEC/WEOWORLD

[29] Gallup International (2017). Religion prevails in the world. https://web.archive.org/web/20171114113506/http://www.wingia.com/web/files/news/370/file/370.pdf

[30] Institute for economics and peace (2023). *Global peace index*. https://www.visionofhumanity.org/wp-content/uploads/2023/06/GPI-2023-Web.pdf *(2023)*

[31] Integrated Values Surveys (2022) – with major processing by Our World in Data. *Most people can be trusted*. https://ourworldindata.org/grapher/self-reported-trust-attitudes

[32] Henrich, J., Heine, S. J. & Norenzayan, A. The weirdest people in the world?. *Behavi. Brain Sci.* **33**(2–3), 61–83. 10.1017/S0140525X0999152X (2010). 10.1017/S0140525X0999152X 20550733

[33] Wong, P. T. & Cowden, R. G. Accelerating the science and practice of psychology beyond WEIRD biases: Enriching the landscape through Asian psychology. *Front. Psychol.* **13**, 1054519. 10.3389/fpsyg.2022.1054519 (2022). 36619071 10.3389/fpsyg.2022.1054519 PMC9815563

[34] Padgett, R. N. et al. Survey sampling design in wave 1 of the Global Flourishing Study. *Eur. J. Epidemiol.* **40**, 391-406. 10.1007/s10654-024-01167-9 (2025). 10.1007/s10654-024-01167-9 PMC12145319 40146468

[35] Ritter, Z. et al. *Global Flourishing Study Methodology* (Gallup Inc., 2024). https://osf.io/k2s7u

[36] Cowden, R. G., Skinstad, D., Lomas, T., Johnson, B. R. & VanderWeele, T. J. Measuring wellbeing in the global flourishing study: Insights from a cross-national analysis of cognitive interviews from 22 countries. *Qual. Quant.* **59**, 575–597. 10.1007/s11135-024-01947-1 (2025).

[37] Lomas, T. et al. The development of the global flourishing study survey: Charting the evolution of a new 109-item inventory of human flourishing. *BMC Global Public Health* **3**, 30. 10.1186/s44263-025-00139-9 (2025). 40307930 10.1186/s44263-025-00139-9 PMC12042518

[38] VanderWeele, T. J. et al. The global flourishing Study: Study profile and initial results on flourishing. *Nat. Mental Health* **3**, 636–653. 10.1038/s44220-025-00423-5 (2025). 40521104 10.1038/s44220-025-00423-5 PMC12165845

[39] Sorgente, A., Zambelli, M., Tagliabue, S. & Lanz, M. The comprehensive inventory of thriving: a systematic review of published validation studies and a replication study. *Curr. Psychol.* **42**, 7920–7937. 10.1007/s12144-021-02065-z (2021).

[40] Little, T. D. *The Oxford handbook of quantitative methods* (Oxford University Press, 2013).

[41] Marsh, H. W., Hau, K. T. & Wen, Z. In search of golden rules: Comment on hypothesis testing approaches to setting cutoff values for fit indexes and dangers in overgeneralising Hu & Bentler’s (1999) findings. *Struct. Equ. Model.* **11**, 320–341. 10.1207/s15328007sem1103_2 (2004).

[42] Borsboom, D. et al. Network analysis of multivariate data in psychological science. *Nat. Rev. Methods Primers* **1**(1), 58. 10.1038/s43586-021-00055-w (2021).

[43] Epskamp, S., Isvoranu, A. M. & Cheung, M. W. L. Meta-analytic Gaussian network aggregation. *Psychometrika* **87**(1), 12–46. 10.1007/s11336-021-09764-3 (2022). 34264449 10.1007/s11336-021-09764-3 PMC9021114

[44] Epskamp, S., Cramer, A. O., Waldorp, L. J., Schmittmann, V. D. & Borsboom, D. Network visualizations of relationships in psychometric data. *J. Stat. Softw.* **48**(1), 18. 10.18637/jss.v048.i04 (2012).

[45] Isvoranu, A. M., Epskamp, S. & Cheung, M. W. L. Network models of posttraumatic stress disorder: A meta-analysis. *J. Abnorm. Psychol.* **130**(8), 841. 10.1037/abn0000704 (2021). 34843289 10.1037/abn0000704

[46] Robinaugh, D. J., Millner, A. J. & McNally, R. J. Identifying highly influential nodes in the complicated grief network. *J. Abnorm. Psychol.* **125**(6), 747–757. 10.1037/abn0000181 (2016). 27505622 10.1037/abn0000181 PMC5060093

[47] Haslbeck, J. M. B. & Waldorp, L. J. How well do network models predict observations? On the importance of predictability in network models. *Behav. Res. Methods* **50**(2), 853–861. 10.3758/s13428-017-0910-x (2018). 28718088 10.3758/s13428-017-0910-x PMC5880858

[48] Jones P. J. *networktools: Assorted tools for identifying important nodes in networks.* R package version 1.2.3. CRAN. https://CRAN.R-project.org/package=networktools (2020).

[49] Curry, O. S. et al. Happy to help? A systematic review and meta-analysis of the effects of performing acts of kindness on the well-being of the actor. *J. Exp. Soc. Psychol.* **76**, 320–329 (2018).

[50] Folk, D. & Dunn, E. How can people become happier? A systematic review of preregistered experiments. *Ann. Rev. Psychol.* **75**(1), 467–493. 10.1146/annurev-psych-022423-030818 (2024). 10.1146/annurev-psych-022423-030818 37566759

[51] Beller, J., Geyer, S. & Epping, J. Health and study dropout: Health aspects differentially predict attrition. *BMC Med. Res. Methodol.* **22**(1), 1–10. 10.1186/s12874-022-01508-w (2022). 35094681 10.1186/s12874-022-01508-w PMC8802529

