# Supplementary material for: The psychometric network of individual flourishing across nationally representative samples from 22 countries
Source: Sci Rep. 2025 Aug 18;15:30206. doi: 10.1038/s41598-025-15016-6 (PMC12361562; doi:10.1038/s41598-025-15016-6)
Supplement: Supplementary file 1 — Supplementary Information. [file 41598_2025_15016_MOESM1_ESM.docx]

**The Psychometric Network of Individual Flourishing across Nationally Representative Samples from 22 Countries**

**Supplementary materials**

Supp. Table S1. Correlations by Country

Supp. Table S2. Expected Influence by Country

Supp. Table S3. Predictability (R2) by Country

**TableS1.** Correlations by Country. The lower diagonal reports the weighted zero-order correlation between the twelve flourishing components. The upper diagonal reports partial correlations obtained from the *single country saturated model* and visualized in Figures 4a-4b.

| Country | Item | H | LS | MH | PH | SP | W | PG | GU | C | SR | E | WS |
| --- | --- | --- | --- | --- | --- | --- | --- | --- | --- | --- | --- | --- | --- |
| Argentina |  |  |  |  |  |  |  |  |  |  |  |  |  |
|  | H | 1.000 | 0.469 | 0.087 | 0.075 | 0.057 | 0.205 | -0.014 | 0.028 | 0.024 | 0.080 | 0.033 | -0.015 |
|  | LS | 0.748 | 1.000 | 0.062 | 0.101 | 0.064 | 0.255 | -0.015 | 0.015 | 0.036 | 0.064 | 0.033 | 0.017 |
|  | MH | 0.525 | 0.518 | 1.000 | 0.287 | 0.180 | 0.087 | 0.111 | 0.045 | 0.062 | 0.052 | 0.011 | 0.022 |
|  | PH | 0.408 | 0.423 | 0.479 | 1.000 | -0.050 | 0.005 | 0.044 | 0.038 | -0.023 | 0.061 | 0.049 | 0.004 |
|  | SP | 0.457 | 0.464 | 0.477 | 0.283 | 1.000 | 0.174 | 0.155 | 0.116 | 0.076 | 0.060 | 0.020 | -0.014 |
|  | W | 0.635 | 0.664 | 0.503 | 0.364 | 0.515 | 1.000 | 0.114 | 0.002 | 0.042 | 0.078 | 0.009 | -0.015 |
|  | PG | 0.277 | 0.274 | 0.342 | 0.229 | 0.376 | 0.351 | 1.000 | 0.225 | 0.037 | 0.020 | -0.024 | -0.029 |
|  | GU | 0.212 | 0.207 | 0.248 | 0.183 | 0.282 | 0.229 | 0.343 | 1.000 | 0.030 | 0.034 | -0.020 | -0.014 |
|  | C | 0.439 | 0.445 | 0.413 | 0.282 | 0.414 | 0.439 | 0.301 | 0.201 | 1.000 | 0.521 | -0.008 | 0.027 |
|  | SR | 0.495 | 0.491 | 0.431 | 0.328 | 0.439 | 0.486 | 0.296 | 0.218 | 0.697 | 1.000 | 0.015 | -0.002 |
|  | E | 0.23 | 0.258 | 0.205 | 0.182 | 0.142 | 0.173 | 0.021 | 0.009 | 0.14 | 0.164 | 1.000 | 0.776 |
|  | WS | 0.176 | 0.201 | 0.158 | 0.148 | 0.094 | 0.128 | -0.003 | -0.008 | 0.113 | 0.122 | 0.793 | 1.000 |
| Australia |  |  |  |  |  |  |  |  |  |  |  |  |  |
|  | H | 1.000 | 0.411 | 0.362 | 0.021 | -0.036 | 0.149 | 0.040 | -0.034 | 0.125 | -0.012 | -0.025 | 0.052 |
|  | LS | 0.817 | 1.000 | 0.141 | 0.048 | 0.064 | 0.294 | -0.069 | 0.039 | -0.025 | 0.108 | 0.079 | 0.038 |
|  | MH | 0.769 | 0.728 | 1.000 | 0.183 | 0.118 | 0.030 | 0.098 | 0.015 | 0.031 | 0.045 | 0.094 | -0.003 |
|  | PH | 0.455 | 0.472 | 0.491 | 1.000 | 0.010 | 0.084 | -0.005 | 0.129 | -0.024 | -0.013 | 0.054 | 0.047 |
|  | SP | 0.568 | 0.58 | 0.579 | 0.36 | 1.000 | 0.283 | 0.121 | 0.083 | 0.130 | -0.028 | 0.047 | -0.002 |
|  | W | 0.705 | 0.727 | 0.643 | 0.443 | 0.646 | 1.000 | 0.094 | 0.031 | 0.016 | 0.054 | -0.063 | 0.021 |
|  | PG | 0.394 | 0.365 | 0.408 | 0.271 | 0.432 | 0.422 | 1.000 | 0.278 | -0.006 | 0.050 | 0.008 | -0.006 |
|  | GU | 0.321 | 0.33 | 0.333 | 0.284 | 0.365 | 0.352 | 0.448 | 1.000 | 0.020 | -0.002 | -0.018 | 0.023 |
|  | C | 0.638 | 0.622 | 0.593 | 0.317 | 0.56 | 0.578 | 0.364 | 0.296 | 1.000 | 0.752 | 0.001 | -0.006 |
|  | SR | 0.624 | 0.622 | 0.577 | 0.315 | 0.525 | 0.571 | 0.355 | 0.285 | 0.861 | 1.000 | -0.015 | 0.031 |
|  | E | 0.416 | 0.455 | 0.444 | 0.336 | 0.332 | 0.35 | 0.217 | 0.193 | 0.336 | 0.328 | 1.000 | 0.705 |
|  | WS | 0.434 | 0.465 | 0.438 | 0.335 | 0.34 | 0.377 | 0.234 | 0.217 | 0.352 | 0.344 | 0.759 | 1.000 |
| Brazil |  |  |  |  |  |  |  |  |  |  |  |  |  |
|  | H | 1.000 | 0.439 | 0.104 | 0.061 | 0.046 | 0.173 | 0.025 | 0.003 | 0.063 | 0.073 | -0.010 | 0.025 |
|  | LS | 0.77 | 1.000 | 0.079 | 0.095 | 0.053 | 0.304 | -0.007 | -0.013 | 0.045 | 0.046 | 0.065 | -0.003 |
|  | MH | 0.584 | 0.585 | 1.000 | 0.264 | 0.155 | 0.100 | 0.116 | 0.000 | 0.048 | 0.051 | 0.013 | 0.042 |
|  | PH | 0.448 | 0.462 | 0.531 | 1.000 | 0.019 | 0.051 | 0.054 | 0.028 | -0.003 | 0.025 | 0.039 | -0.001 |
|  | SP | 0.527 | 0.533 | 0.53 | 0.376 | 1.000 | 0.169 | 0.154 | 0.120 | 0.090 | 0.065 | 0.046 | -0.032 |
|  | W | 0.679 | 0.716 | 0.571 | 0.433 | 0.575 | 1.000 | 0.056 | 0.042 | 0.041 | 0.063 | -0.004 | 0.006 |
|  | PG | 0.344 | 0.331 | 0.376 | 0.305 | 0.422 | 0.378 | 1.000 | 0.330 | 0.055 | -0.020 | -0.029 | -0.044 |
|  | GU | 0.271 | 0.274 | 0.274 | 0.234 | 0.365 | 0.306 | 0.448 | 1.000 | 0.045 | 0.012 | 0.015 | -0.034 |
|  | C | 0.535 | 0.525 | 0.47 | 0.345 | 0.497 | 0.521 | 0.333 | 0.281 | 1.000 | 0.607 | -0.023 | 0.020 |
|  | SR | 0.54 | 0.535 | 0.474 | 0.347 | 0.49 | 0.524 | 0.308 | 0.265 | 0.755 | 1.000 | 0.029 | -0.007 |
|  | E | 0.234 | 0.262 | 0.234 | 0.194 | 0.181 | 0.206 | 0.027 | 0.052 | 0.159 | 0.188 | 1.000 | 0.743 |
|  | WS | 0.192 | 0.211 | 0.2 | 0.16 | 0.124 | 0.163 | -0.005 | 0.011 | 0.136 | 0.155 | 0.77 | 1.000 |
| Egypt |  |  |  |  |  |  |  |  |  |  |  |  |  |
|  | H | 1.000 | 0.255 | 0.070 | 0.165 | 0.020 | 0.090 | -0.026 | 0.017 | 0.039 | 0.066 | 0.098 | 0.085 |
|  | LS | 0.424 | 1.000 | 0.042 | 0.066 | 0.045 | 0.217 | 0.049 | 0.031 | 0.031 | 0.003 | 0.043 | 0.088 |
|  | MH | 0.236 | 0.242 | 1.000 | 0.148 | 0.113 | 0.078 | 0.146 | 0.050 | 0.102 | 0.001 | -0.025 | 0.015 |
|  | PH | 0.331 | 0.263 | 0.273 | 1.000 | 0.042 | 0.016 | 0.003 | 0.063 | 0.022 | 0.037 | 0.103 | 0.048 |
|  | SP | 0.223 | 0.253 | 0.267 | 0.194 | 1.000 | 0.193 | 0.092 | 0.081 | 0.109 | 0.127 | 0.002 | 0.031 |
|  | W | 0.275 | 0.368 | 0.258 | 0.194 | 0.341 | 1.000 | 0.092 | 0.035 | 0.065 | 0.029 | 0.023 | 0.000 |
|  | PG | 0.103 | 0.166 | 0.239 | 0.121 | 0.257 | 0.234 | 1.000 | 0.143 | 0.032 | 0.068 | -0.014 | -0.007 |
|  | GU | 0.113 | 0.131 | 0.148 | 0.137 | 0.189 | 0.153 | 0.206 | 1.000 | 0.041 | 0.001 | -0.030 | 0.033 |
|  | C | 0.241 | 0.23 | 0.242 | 0.196 | 0.302 | 0.248 | 0.197 | 0.154 | 1.000 | 0.408 | 0.051 | 0.002 |
|  | SR | 0.239 | 0.2 | 0.198 | 0.194 | 0.29 | 0.223 | 0.198 | 0.12 | 0.52 | 1.000 | 0.047 | 0.033 |
|  | E | 0.32 | 0.261 | 0.133 | 0.258 | 0.15 | 0.173 | 0.067 | 0.07 | 0.18 | 0.192 | 1.000 | 0.539 |
|  | WS | 0.314 | 0.273 | 0.152 | 0.243 | 0.169 | 0.183 | 0.09 | 0.076 | 0.191 | 0.197 | 0.605 | 1.000 |
| Germany |  |  |  |  |  |  |  |  |  |  |  |  |  |
|  | H | 1.000 | 0.655 | 0.141 | 0.004 | 0.037 | 0.074 | 0.021 | 0.025 | -0.021 | 0.097 | 0.005 | 0.006 |
|  | LS | 0.835 | 1.000 | 0.028 | 0.061 | -0.018 | 0.355 | -0.043 | 0.002 | 0.015 | 0.026 | 0.085 | 0.022 |
|  | MH | 0.51 | 0.49 | 1.000 | 0.472 | 0.150 | 0.013 | 0.060 | -0.013 | 0.043 | 0.041 | 0.041 | 0.022 |
|  | PH | 0.373 | 0.387 | 0.587 | 1.000 | -0.033 | -0.035 | -0.008 | 0.076 | -0.026 | 0.019 | 0.048 | 0.022 |
|  | SP | 0.412 | 0.403 | 0.401 | 0.25 | 1.000 | 0.208 | 0.135 | 0.099 | 0.101 | 0.017 | 0.028 | 0.011 |
|  | W | 0.627 | 0.686 | 0.403 | 0.278 | 0.455 | 1.000 | 0.119 | 0.027 | 0.054 | -0.009 | -0.048 | 0.034 |
|  | PG | 0.232 | 0.229 | 0.23 | 0.164 | 0.302 | 0.296 | 1.000 | 0.185 | 0.050 | -0.006 | -0.017 | 0.023 |
|  | GU | 0.216 | 0.213 | 0.198 | 0.201 | 0.248 | 0.217 | 0.285 | 1.000 | -0.007 | 0.033 | 0.013 | 0.000 |
|  | C | 0.419 | 0.409 | 0.363 | 0.228 | 0.37 | 0.38 | 0.239 | 0.182 | 1.000 | 0.737 | -0.021 | 0.042 |
|  | SR | 0.456 | 0.433 | 0.37 | 0.257 | 0.353 | 0.381 | 0.213 | 0.184 | 0.805 | 1.000 | 0.014 | -0.020 |
|  | E | 0.338 | 0.372 | 0.318 | 0.28 | 0.227 | 0.259 | 0.115 | 0.127 | 0.209 | 0.223 | 1.000 | 0.700 |
|  | WS | 0.326 | 0.351 | 0.301 | 0.265 | 0.229 | 0.267 | 0.132 | 0.13 | 0.22 | 0.224 | 0.757 | 1.000 |
| Hong Kong |  |  |  |  |  |  |  |  |  |  |  |  |  |
|  | H | 1.000 | 0.383 | 0.141 | 0.166 | 0.028 | 0.240 | 0.158 | -0.026 | 0.007 | 0.030 | 0.086 | -0.028 |
|  | LS | 0.854 | 1.000 | 0.149 | 0.054 | 0.056 | 0.226 | -0.051 | 0.016 | 0.035 | 0.078 | 0.073 | 0.064 |
|  | MH | 0.800 | 0.802 | 1.000 | 0.218 | -0.023 | 0.055 | 0.079 | 0.038 | 0.112 | 0.111 | 0.031 | 0.025 |
|  | PH | 0.783 | 0.744 | 0.767 | 1.000 | 0.076 | 0.005 | 0.167 | 0.041 | 0.002 | 0.052 | 0.048 | 0.031 |
|  | SP | 0.732 | 0.706 | 0.692 | 0.709 | 1.000 | 0.108 | 0.239 | 0.112 | 0.098 | 0.114 | 0.093 | -0.041 |
|  | W | 0.802 | 0.808 | 0.768 | 0.714 | 0.714 | 1.000 | 0.110 | 0.109 | 0.054 | 0.039 | -0.026 | 0.061 |
|  | PG | 0.777 | 0.720 | 0.738 | 0.753 | 0.760 | 0.735 | 1.000 | 0.301 | 0.038 | 0.029 | 0.036 | -0.027 |
|  | GU | 0.632 | 0.630 | 0.655 | 0.630 | 0.653 | 0.641 | 0.701 | 1.000 | 0.063 | -0.012 | -0.031 | 0.056 |
|  | C | 0.723 | 0.734 | 0.748 | 0.689 | 0.703 | 0.734 | 0.703 | 0.626 | 1.000 | 0.553 | 0.028 | 0.038 |
|  | SR | 0.736 | 0.735 | 0.739 | 0.693 | 0.712 | 0.737 | 0.702 | 0.616 | 0.833 | 1.000 | -0.038 | 0.010 |
|  | E | 0.619 | 0.622 | 0.588 | 0.592 | 0.543 | 0.579 | 0.559 | 0.492 | 0.538 | 0.537 | 1.000 | 0.580 |
|  | WS | 0.570 | 0.580 | 0.548 | 0.542 | 0.485 | 0.562 | 0.506 | 0.461 | 0.516 | 0.505 | 0.739 | 1.000 |
| India |  |  |  |  |  |  |  |  |  |  |  |  |  |
|  | H | 1.000 | 0.386 | 0.077 | 0.103 | 0.007 | 0.134 | 0.024 | 0.045 | 0.041 | 0.016 | 0.044 | 0.034 |
|  | LS | 0.574 | 1.000 | 0.117 | 0.046 | 0.031 | 0.229 | 0.038 | 0.033 | 0.038 | 0.068 | -0.001 | 0.029 |
|  | MH | 0.39 | 0.425 | 1.000 | 0.373 | 0.086 | 0.069 | 0.134 | -0.004 | 0.078 | 0.014 | 0.016 | 0.000 |
|  | PH | 0.39 | 0.386 | 0.551 | 1.000 | 0.040 | 0.118 | 0.026 | 0.041 | 0.013 | 0.024 | 0.042 | 0.017 |
|  | SP | 0.248 | 0.296 | 0.339 | 0.288 | 1.000 | 0.092 | 0.198 | 0.102 | 0.125 | 0.073 | -0.013 | -0.002 |
|  | W | 0.445 | 0.498 | 0.393 | 0.392 | 0.319 | 1.000 | 0.077 | 0.034 | 0.037 | 0.041 | 0.062 | 0.022 |
|  | PG | 0.269 | 0.311 | 0.368 | 0.302 | 0.389 | 0.324 | 1.000 | 0.166 | 0.050 | 0.089 | -0.025 | -0.015 |
|  | GU | 0.22 | 0.224 | 0.222 | 0.217 | 0.275 | 0.23 | 0.316 | 1.000 | 0.044 | 0.034 | 0.024 | -0.016 |
|  | C | 0.269 | 0.317 | 0.321 | 0.265 | 0.355 | 0.296 | 0.308 | 0.22 | 1.000 | 0.435 | 0.037 | -0.019 |
|  | SR | 0.261 | 0.312 | 0.286 | 0.249 | 0.336 | 0.29 | 0.315 | 0.213 | 0.54 | 1.000 | -0.023 | -0.022 |
|  | E | 0.192 | 0.157 | 0.139 | 0.161 | 0.061 | 0.177 | 0.049 | 0.07 | 0.08 | 0.045 | 1.000 | 0.568 |
|  | WS | 0.16 | 0.137 | 0.099 | 0.12 | 0.039 | 0.139 | 0.022 | 0.038 | 0.039 | 0.014 | 0.578 | 1.000 |
| Indonesia |  |  |  |  |  |  |  |  |  |  |  |  |  |
|  | H | 1.000 | 0.304 | 0.136 | 0.108 | -0.017 | 0.167 | 0.009 | 0.041 | 0.014 | 0.048 | 0.058 | 0.028 |
|  | LS | 0.615 | 1.000 | 0.036 | 0.132 | 0.026 | 0.265 | -0.002 | 0.048 | -0.010 | 0.093 | 0.097 | -0.005 |
|  | MH | 0.469 | 0.462 | 1.000 | 0.294 | 0.051 | 0.120 | 0.078 | 0.072 | 0.078 | 0.083 | -0.012 | 0.055 |
|  | PH | 0.436 | 0.464 | 0.563 | 1.000 | 0.050 | 0.038 | 0.097 | 0.043 | 0.041 | 0.025 | 0.032 | 0.000 |
|  | SP | 0.341 | 0.363 | 0.431 | 0.385 | 1.000 | 0.132 | 0.113 | 0.102 | 0.186 | 0.179 | 0.008 | -0.019 |
|  | W | 0.524 | 0.559 | 0.491 | 0.439 | 0.43 | 1.000 | 0.118 | 0.061 | 0.024 | 0.023 | -0.009 | -0.020 |
|  | PG | 0.348 | 0.365 | 0.44 | 0.406 | 0.459 | 0.445 | 1.000 | 0.316 | 0.116 | 0.060 | -0.007 | 0.011 |
|  | GU | 0.329 | 0.338 | 0.38 | 0.358 | 0.392 | 0.387 | 0.515 | 1.000 | 0.037 | 0.019 | 0.007 | 0.003 |
|  | C | 0.359 | 0.37 | 0.442 | 0.376 | 0.505 | 0.402 | 0.416 | 0.342 | 1.000 | 0.389 | -0.010 | -0.015 |
|  | SR | 0.41 | 0.438 | 0.455 | 0.374 | 0.498 | 0.405 | 0.422 | 0.352 | 0.614 | 1.000 | 0.019 | 0.030 |
|  | E | 0.243 | 0.273 | 0.191 | 0.187 | 0.138 | 0.18 | 0.139 | 0.129 | 0.125 | 0.183 | 1.000 | 0.605 |
|  | WS | 0.2 | 0.208 | 0.166 | 0.153 | 0.114 | 0.14 | 0.123 | 0.109 | 0.111 | 0.166 | 0.642 | 1.000 |
| Israel |  |  |  |  |  |  |  |  |  |  |  |  |  |
|  | H | 1.000 | 0.358 | 0.170 | 0.043 | 0.022 | 0.246 | 0.018 | -0.007 | 0.017 | 0.113 | 0.098 | 0.021 |
|  | LS | 0.662 | 1.000 | 0.091 | 0.050 | -0.024 | 0.102 | -0.004 | 0.067 | 0.064 | -0.026 | -0.055 | 0.116 |
|  | MH | 0.595 | 0.52 | 1.000 | 0.393 | 0.107 | 0.011 | 0.125 | -0.037 | 0.022 | 0.017 | 0.116 | -0.017 |
|  | PH | 0.466 | 0.397 | 0.609 | 1.000 | 0.055 | 0.045 | 0.056 | -0.003 | 0.042 | -0.024 | 0.026 | -0.016 |
|  | SP | 0.504 | 0.425 | 0.502 | 0.398 | 1.000 | 0.293 | 0.163 | 0.083 | 0.102 | 0.018 | -0.004 | 0.060 |
|  | W | 0.644 | 0.552 | 0.528 | 0.436 | 0.632 | 1.000 | 0.086 | 0.079 | 0.117 | 0.004 | 0.092 | 0.011 |
|  | PG | 0.416 | 0.359 | 0.44 | 0.361 | 0.491 | 0.471 | 1.000 | 0.239 | 0.046 | 0.001 | 0.028 | -0.010 |
|  | GU | 0.249 | 0.231 | 0.222 | 0.192 | 0.309 | 0.305 | 0.377 | 1.000 | -0.069 | 0.111 | -0.007 | -0.019 |
|  | C | 0.531 | 0.464 | 0.462 | 0.382 | 0.52 | 0.561 | 0.4 | 0.261 | 1.000 | 0.750 | -0.095 | 0.044 |
|  | SR | 0.535 | 0.438 | 0.449 | 0.353 | 0.491 | 0.532 | 0.382 | 0.288 | 0.846 | 1.000 | 0.067 | -0.002 |
|  | E | 0.474 | 0.382 | 0.448 | 0.332 | 0.394 | 0.447 | 0.304 | 0.165 | 0.341 | 0.37 | 1.000 | 0.678 |
|  | WS | 0.456 | 0.407 | 0.407 | 0.296 | 0.385 | 0.433 | 0.272 | 0.16 | 0.345 | 0.359 | 0.779 | 1.000 |
| Japan |  |  |  |  |  |  |  |  |  |  |  |  |  |
|  | HA | 1.000 | 0.589 | 0.096 | 0.041 | 0.000 | 0.163 | 0.084 | -0.040 | 0.013 | 0.050 | -0.023 | 0.016 |
|  | LS | 0.887 | 1.000 | 0.158 | 0.001 | -0.015 | 0.220 | -0.050 | 0.013 | 0.010 | 0.059 | 0.147 | 0.030 |
|  | MH | 0.755 | 0.763 | 1.000 | 0.445 | 0.030 | 0.051 | 0.178 | -0.021 | 0.066 | 0.053 | -0.018 | 0.056 |
|  | PH | 0.610 | 0.610 | 0.737 | 1.000 | -0.024 | 0.036 | 0.049 | 0.082 | -0.002 | 0.000 | 0.008 | 0.023 |
|  | LP | 0.639 | 0.639 | 0.641 | 0.507 | 1.000 | 0.256 | 0.264 | 0.192 | 0.099 | 0.066 | 0.049 | -0.024 |
|  | W | 0.785 | 0.791 | 0.723 | 0.584 | 0.722 | 1.000 | 0.149 | 0.011 | 0.029 | 0.029 | -0.035 | 0.024 |
|  | PG | 0.667 | 0.653 | 0.699 | 0.563 | 0.713 | 0.708 | 1.000 | 0.172 | 0.043 | 0.041 | -0.008 | 0.021 |
|  | GU | 0.384 | 0.394 | 0.412 | 0.370 | 0.506 | 0.435 | 0.511 | 1.000 | -0.002 | 0.018 | 0.050 | -0.025 |
|  | C | 0.684 | 0.691 | 0.689 | 0.541 | 0.667 | 0.683 | 0.654 | 0.405 | 1.000 | 0.738 | -0.033 | 0.047 |
|  | SR | 0.702 | 0.713 | 0.699 | 0.551 | 0.670 | 0.692 | 0.661 | 0.411 | 0.906 | 1.000 | 0.003 | 0.024 |
|  | E | 0.526 | 0.584 | 0.505 | 0.414 | 0.437 | 0.485 | 0.437 | 0.304 | 0.454 | 0.470 | 1.000 | 0.700 |
|  | WS | 0.555 | 0.600 | 0.548 | 0.454 | 0.454 | 0.520 | 0.470 | 0.297 | 0.503 | 0.514 | 0.809 | 1.000 |
| Kenya |  |  |  |  |  |  |  |  |  |  |  |  |  |
|  | HA | 1.000 | 0.203 | 0.061 | 0.147 | 0.036 | 0.126 | 0.030 | 0.027 | 0.040 | 0.072 | 0.048 | 0.050 |
|  | LS | 0.350 | 1.000 | -0.003 | 0.032 | -0.002 | 0.206 | 0.022 | 0.007 | 0.009 | 0.076 | 0.123 | 0.084 |
|  | MH | 0.255 | 0.163 | 1.000 | 0.260 | 0.122 | 0.084 | 0.042 | 0.079 | 0.093 | 0.046 | -0.027 | -0.002 |
|  | PH | 0.326 | 0.231 | 0.399 | 1.000 | 0.056 | 0.133 | 0.075 | 0.043 | 0.031 | 0.010 | 0.050 | 0.020 |
|  | LP | 0.206 | 0.149 | 0.292 | 0.252 | 1.000 | 0.039 | 0.154 | 0.114 | 0.137 | 0.090 | 0.009 | -0.005 |
|  | W | 0.323 | 0.352 | 0.263 | 0.317 | 0.235 | 1.000 | 0.066 | 0.056 | 0.034 | 0.057 | 0.013 | 0.042 |
|  | PG | 0.209 | 0.158 | 0.228 | 0.241 | 0.312 | 0.241 | 1.000 | 0.226 | 0.077 | 0.054 | -0.021 | 0.009 |
|  | GU | 0.195 | 0.134 | 0.236 | 0.233 | 0.281 | 0.212 | 0.341 | 1.000 | 0.070 | 0.043 | -0.004 | 0.001 |
|  | C | 0.232 | 0.177 | 0.258 | 0.227 | 0.321 | 0.225 | 0.261 | 0.250 | 1.000 | 0.334 | 0.003 | 0.007 |
|  | SR | 0.246 | 0.232 | 0.226 | 0.222 | 0.271 | 0.247 | 0.250 | 0.229 | 0.460 | 1.000 | 0.061 | 0.025 |
|  | E | 0.204 | 0.280 | 0.081 | 0.153 | 0.084 | 0.176 | 0.073 | 0.073 | 0.112 | 0.176 | 1.000 | 0.504 |
|  | WS | 0.205 | 0.274 | 0.087 | 0.148 | 0.084 | 0.182 | 0.083 | 0.077 | 0.123 | 0.163 | 0.544 | 1.000 |
| Mexico |  |  |  |  |  |  |  |  |  |  |  |  |  |
|  | HA | 1.000 | 0.461 | 0.096 | 0.088 | 0.015 | 0.162 | 0.037 | 0.013 | -0.010 | 0.063 | 0.023 | 0.017 |
|  | LS | 0.742 | 1.000 | 0.072 | 0.104 | 0.022 | 0.292 | -0.025 | -0.027 | 0.060 | 0.106 | -0.008 | 0.041 |
|  | MH | 0.528 | 0.549 | 1.000 | 0.288 | 0.162 | 0.121 | 0.127 | 0.009 | 0.052 | 0.065 | 0.012 | -0.006 |
|  | PH | 0.466 | 0.491 | 0.552 | 1.000 | 0.016 | 0.033 | 0.091 | 0.043 | -0.032 | 0.034 | 0.034 | 0.010 |
|  | LP | 0.458 | 0.475 | 0.534 | 0.376 | 1.000 | 0.197 | 0.151 | 0.089 | 0.123 | 0.074 | 0.011 | -0.007 |
|  | W | 0.622 | 0.677 | 0.555 | 0.436 | 0.538 | 1.000 | 0.039 | 0.015 | 0.101 | -0.015 | 0.025 | -0.030 |
|  | PG | 0.348 | 0.361 | 0.437 | 0.359 | 0.456 | 0.398 | 1.000 | 0.272 | 0.071 | 0.048 | -0.021 | -0.038 |
|  | GU | 0.264 | 0.265 | 0.319 | 0.262 | 0.357 | 0.293 | 0.441 | 1.000 | 0.052 | 0.035 | -0.003 | 0.011 |
|  | C | 0.450 | 0.491 | 0.473 | 0.350 | 0.491 | 0.491 | 0.385 | 0.303 | 1.000 | 0.490 | 0.005 | 0.004 |
|  | SR | 0.495 | 0.532 | 0.479 | 0.372 | 0.485 | 0.497 | 0.373 | 0.285 | 0.690 | 1.000 | 0.058 | 0.001 |
|  | E | 0.218 | 0.227 | 0.177 | 0.181 | 0.144 | 0.182 | 0.069 | 0.067 | 0.188 | 0.223 | 1.000 | 0.750 |
|  | WS | 0.189 | 0.197 | 0.150 | 0.159 | 0.108 | 0.140 | 0.047 | 0.051 | 0.152 | 0.187 | 0.777 | 1.000 |
| Nigeria |  |  |  |  |  |  |  |  |  |  |  |  |  |
|  | HA | 1.000 | 0.353 | 0.015 | 0.101 | 0.059 | 0.066 | 0.017 | 0.048 | 0.031 | 0.057 | 0.046 | 0.031 |
|  | LS | 0.506 | 1.000 | 0.025 | 0.013 | -0.045 | 0.245 | 0.004 | 0.035 | 0.022 | 0.095 | 0.122 | 0.060 |
|  | MH | 0.262 | 0.217 | 1.000 | 0.329 | 0.141 | 0.087 | 0.047 | 0.121 | 0.039 | 0.005 | -0.047 | 0.022 |
|  | PH | 0.294 | 0.246 | 0.528 | 1.000 | 0.049 | 0.157 | 0.111 | 0.086 | 0.007 | 0.017 | 0.007 | 0.002 |
|  | LP | 0.253 | 0.187 | 0.376 | 0.354 | 1.000 | 0.135 | 0.125 | 0.147 | 0.167 | 0.044 | -0.029 | 0.006 |
|  | W | 0.354 | 0.437 | 0.357 | 0.383 | 0.317 | 1.000 | 0.048 | 0.005 | 0.041 | 0.001 | 0.031 | 0.037 |
|  | PG | 0.247 | 0.200 | 0.357 | 0.367 | 0.400 | 0.277 | 1.000 | 0.353 | 0.047 | 0.083 | 0.040 | -0.081 |
|  | GU | 0.236 | 0.196 | 0.376 | 0.376 | 0.399 | 0.282 | 0.544 | 1.000 | 0.045 | 0.068 | -0.077 | 0.028 |
|  | C | 0.260 | 0.233 | 0.285 | 0.292 | 0.384 | 0.268 | 0.315 | 0.317 | 1.000 | 0.474 | 0.043 | -0.017 |
|  | SR | 0.280 | 0.284 | 0.266 | 0.269 | 0.328 | 0.261 | 0.323 | 0.315 | 0.587 | 1.000 | 0.049 | -0.005 |
|  | E | 0.226 | 0.310 | 0.032 | 0.056 | 0.037 | 0.185 | 0.029 | -0.010 | 0.103 | 0.158 | 1.000 | 0.587 |
|  | WS | 0.207 | 0.277 | 0.056 | 0.063 | 0.044 | 0.182 | 0.001 | 0.011 | 0.074 | 0.106 | 0.640 | 1.000 |
| Philippines |  |  |  |  |  |  |  |  |  |  |  |  |  |
|  | HA | 1.000 | 0.387 | 0.092 | 0.137 | -0.011 | 0.055 | -0.009 | 0.033 | 0.037 | 0.084 | 0.032 | 0.054 |
|  | LS | 0.592 | 1.000 | 0.109 | 0.077 | -0.030 | 0.161 | 0.017 | 0.028 | 0.060 | 0.057 | 0.065 | 0.039 |
|  | MH | 0.394 | 0.414 | 1.000 | 0.331 | 0.115 | 0.101 | 0.063 | 0.078 | 0.026 | 0.026 | -0.007 | 0.027 |
|  | PH | 0.394 | 0.388 | 0.536 | 1.000 | 0.050 | 0.043 | 0.063 | 0.006 | 0.050 | 0.012 | 0.045 | 0.000 |
|  | LP | 0.292 | 0.314 | 0.424 | 0.346 | 1.000 | 0.148 | 0.103 | 0.144 | 0.208 | 0.108 | -0.013 | 0.033 |
|  | W | 0.362 | 0.430 | 0.410 | 0.337 | 0.412 | 1.000 | 0.143 | 0.047 | 0.026 | 0.037 | 0.017 | 0.007 |
|  | PG | 0.282 | 0.315 | 0.409 | 0.332 | 0.453 | 0.393 | 1.000 | 0.396 | 0.037 | 0.075 | 0.005 | -0.026 |
|  | GU | 0.307 | 0.326 | 0.394 | 0.323 | 0.442 | 0.366 | 0.576 | 1.000 | 0.036 | 0.102 | -0.021 | 0.033 |
|  | C | 0.344 | 0.369 | 0.376 | 0.313 | 0.486 | 0.350 | 0.396 | 0.395 | 1.000 | 0.441 | -0.011 | 0.005 |
|  | SR | 0.368 | 0.383 | 0.380 | 0.318 | 0.455 | 0.372 | 0.398 | 0.415 | 0.626 | 1.000 | 0.023 | -0.008 |
|  | E | 0.227 | 0.241 | 0.147 | 0.165 | 0.101 | 0.150 | 0.083 | 0.096 | 0.119 | 0.132 | 1.000 | 0.578 |
|  | WS | 0.243 | 0.250 | 0.185 | 0.190 | 0.147 | 0.164 | 0.112 | 0.139 | 0.150 | 0.153 | 0.606 | 1.000 |
| Poland |  |  |  |  |  |  |  |  |  |  |  |  |  |
|  | HA | 1.000 | 0.442 | 0.037 | 0.199 | -0.013 | 0.181 | 0.055 | 0.003 | 0.064 | 0.074 | 0.052 | -0.002 |
|  | LS | 0.772 | 1.000 | 0.180 | 0.027 | -0.003 | 0.203 | -0.016 | 0.028 | 0.042 | 0.081 | 0.032 | 0.012 |
|  | MH | 0.585 | 0.594 | 1.000 | 0.387 | 0.060 | 0.080 | 0.092 | -0.114 | 0.055 | 0.058 | -0.047 | 0.085 |
|  | PH | 0.553 | 0.522 | 0.668 | 1.000 | -0.059 | -0.004 | 0.069 | -0.017 | 0.004 | -0.007 | 0.094 | -0.004 |
|  | LP | 0.453 | 0.451 | 0.411 | 0.321 | 1.000 | 0.291 | 0.118 | 0.179 | 0.140 | 0.074 | 0.022 | 0.025 |
|  | W | 0.619 | 0.639 | 0.533 | 0.437 | 0.574 | 1.000 | 0.175 | 0.005 | -0.016 | 0.044 | 0.031 | 0.023 |
|  | PG | 0.465 | 0.442 | 0.452 | 0.397 | 0.446 | 0.504 | 1.000 | 0.180 | 0.079 | 0.013 | -0.045 | 0.041 |
|  | GU | 0.188 | 0.180 | 0.083 | 0.072 | 0.338 | 0.226 | 0.282 | 1.000 | 0.005 | 0.070 | 0.007 | -0.026 |
|  | C | 0.566 | 0.558 | 0.501 | 0.401 | 0.519 | 0.508 | 0.463 | 0.241 | 1.000 | 0.612 | 0.003 | 0.063 |
|  | SR | 0.566 | 0.554 | 0.488 | 0.398 | 0.494 | 0.503 | 0.436 | 0.253 | 0.778 | 1.000 | -0.018 | 0.001 |
|  | E | 0.393 | 0.397 | 0.368 | 0.333 | 0.301 | 0.354 | 0.248 | 0.092 | 0.326 | 0.313 | 1.000 | 0.739 |
|  | WS | 0.399 | 0.415 | 0.406 | 0.336 | 0.330 | 0.387 | 0.294 | 0.096 | 0.369 | 0.348 | 0.786 | 1.000 |
| South Africa |  |  |  |  |  |  |  |  |  |  |  |  |  |
|  | HA | 1.000 | 0.303 | 0.059 | 0.068 | 0.038 | 0.093 | 0.016 | 0.073 | 0.058 | 0.090 | 0.064 | 0.085 |
|  | LS | 0.505 | 1.000 | 0.016 | 0.014 | -0.002 | 0.273 | 0.054 | 0.008 | 0.021 | 0.132 | 0.081 | 0.064 |
|  | MH | 0.260 | 0.218 | 1.000 | 0.373 | 0.114 | 0.002 | 0.098 | 0.036 | 0.062 | 0.025 | -0.010 | -0.017 |
|  | PH | 0.289 | 0.244 | 0.492 | 1.000 | 0.051 | 0.139 | 0.043 | 0.087 | -0.043 | 0.023 | 0.037 | -0.039 |
|  | LP | 0.289 | 0.262 | 0.318 | 0.284 | 1.000 | 0.076 | 0.171 | 0.159 | 0.183 | 0.019 | -0.022 | -0.003 |
|  | W | 0.411 | 0.467 | 0.251 | 0.299 | 0.309 | 1.000 | 0.184 | -0.007 | -0.012 | 0.054 | 0.045 | 0.039 |
|  | PG | 0.286 | 0.263 | 0.320 | 0.291 | 0.393 | 0.347 | 1.000 | 0.258 | 0.020 | -0.018 | 0.008 | -0.024 |
|  | GU | 0.303 | 0.243 | 0.265 | 0.261 | 0.351 | 0.267 | 0.412 | 1.000 | 0.117 | 0.046 | -0.052 | 0.036 |
|  | C | 0.334 | 0.306 | 0.236 | 0.204 | 0.376 | 0.275 | 0.280 | 0.317 | 1.000 | 0.454 | 0.004 | 0.004 |
|  | SR | 0.348 | 0.354 | 0.213 | 0.216 | 0.288 | 0.294 | 0.252 | 0.272 | 0.561 | 1.000 | 0.031 | 0.033 |
|  | E | 0.244 | 0.272 | 0.050 | 0.109 | 0.088 | 0.217 | 0.078 | 0.076 | 0.103 | 0.170 | 1.000 | 0.572 |
|  | WS | 0.238 | 0.275 | 0.066 | 0.088 | 0.083 | 0.201 | 0.071 | 0.095 | 0.117 | 0.178 | 0.611 | 1.000 |
| Spain |  |  |  |  |  |  |  |  |  |  |  |  |  |
|  | HA | 1.000 | 0.536 | 0.087 | 0.055 | 0.011 | 0.255 | 0.029 | -0.003 | 0.012 | 0.098 | 0.032 | -0.039 |
|  | LS | 0.814 | 1.000 | 0.029 | 0.050 | 0.027 | 0.380 | -0.031 | -0.030 | 0.015 | 0.040 | 0.049 | 0.053 |
|  | MH | 0.506 | 0.497 | 1.000 | 0.409 | 0.093 | 0.075 | 0.086 | 0.040 | 0.050 | 0.053 | 0.023 | 0.030 |
|  | PH | 0.405 | 0.391 | 0.578 | 1.000 | 0.058 | -0.038 | 0.022 | 0.056 | 0.002 | 0.015 | 0.019 | 0.017 |
|  | LP | 0.443 | 0.439 | 0.430 | 0.332 | 1.000 | 0.177 | 0.108 | 0.118 | 0.075 | 0.091 | 0.012 | 0.005 |
|  | W | 0.745 | 0.763 | 0.491 | 0.370 | 0.496 | 1.000 | 0.037 | 0.050 | 0.051 | -0.025 | -0.046 | 0.007 |
|  | PG | 0.279 | 0.259 | 0.299 | 0.229 | 0.325 | 0.307 | 1.000 | 0.273 | 0.111 | -0.004 | -0.040 | -0.017 |
|  | GU | 0.236 | 0.221 | 0.263 | 0.213 | 0.308 | 0.275 | 0.371 | 1.000 | 0.023 | 0.012 | 0.018 | -0.016 |
|  | C | 0.440 | 0.431 | 0.405 | 0.296 | 0.411 | 0.429 | 0.300 | 0.225 | 1.000 | 0.616 | -0.027 | 0.036 |
|  | SR | 0.471 | 0.461 | 0.415 | 0.306 | 0.411 | 0.428 | 0.264 | 0.220 | 0.737 | 1.000 | 0.042 | -0.015 |
|  | E | 0.230 | 0.265 | 0.211 | 0.169 | 0.147 | 0.186 | 0.015 | 0.044 | 0.151 | 0.182 | 1.000 | 0.696 |
|  | WS | 0.190 | 0.236 | 0.193 | 0.154 | 0.123 | 0.168 | 0.012 | 0.024 | 0.143 | 0.158 | 0.711 | 1.000 |
| Tanzania |  |  |  |  |  |  |  |  |  |  |  |  |  |
|  | HA | 1.000 | 0.261 | 0.035 | 0.154 | 0.027 | 0.168 | 0.042 | 0.012 | 0.029 | 0.034 | 0.059 | 0.095 |
|  | LS | 0.458 | 1.000 | -0.029 | 0.007 | -0.034 | 0.218 | -0.030 | 0.005 | 0.027 | 0.088 | 0.115 | 0.085 |
|  | MH | 0.251 | 0.155 | 1.000 | 0.273 | 0.101 | 0.090 | 0.114 | 0.045 | 0.075 | 0.018 | -0.009 | 0.001 |
|  | PH | 0.370 | 0.255 | 0.413 | 1.000 | 0.066 | 0.133 | 0.078 | 0.065 | -0.006 | 0.034 | 0.063 | 0.042 |
|  | LP | 0.253 | 0.186 | 0.305 | 0.294 | 1.000 | 0.143 | 0.138 | 0.100 | 0.125 | 0.080 | -0.021 | 0.055 |
|  | W | 0.422 | 0.406 | 0.314 | 0.395 | 0.341 | 1.000 | 0.069 | 0.059 | 0.028 | 0.030 | 0.030 | 0.035 |
|  | PG | 0.226 | 0.162 | 0.312 | 0.295 | 0.347 | 0.296 | 1.000 | 0.225 | 0.099 | 0.032 | -0.021 | 0.040 |
|  | GU | 0.199 | 0.163 | 0.223 | 0.244 | 0.288 | 0.259 | 0.359 | 1.000 | 0.049 | 0.027 | 0.032 | -0.002 |
|  | C | 0.255 | 0.235 | 0.246 | 0.239 | 0.326 | 0.270 | 0.292 | 0.236 | 1.000 | 0.410 | 0.078 | -0.020 |
|  | SR | 0.262 | 0.272 | 0.217 | 0.241 | 0.292 | 0.280 | 0.234 | 0.198 | 0.522 | 1.000 | 0.031 | 0.027 |
|  | E | 0.327 | 0.339 | 0.141 | 0.252 | 0.172 | 0.272 | 0.157 | 0.152 | 0.230 | 0.224 | 1.000 | 0.475 |
|  | WS | 0.314 | 0.324 | 0.158 | 0.246 | 0.207 | 0.289 | 0.184 | 0.148 | 0.195 | 0.209 | 0.555 | 1.000 |
| Turkey |  |  |  |  |  |  |  |  |  |  |  |  |  |
|  | HA | 1.000 | 0.539 | 0.108 | 0.044 | 0.019 | 0.209 | 0.053 | -0.059 | -0.004 | 0.061 | -0.002 | 0.027 |
|  | LS | 0.774 | 1.000 | 0.157 | -0.050 | -0.001 | 0.147 | -0.037 | 0.052 | 0.000 | 0.041 | 0.167 | 0.093 |
|  | MH | 0.551 | 0.558 | 1.000 | 0.337 | 0.134 | 0.082 | -0.036 | 0.005 | 0.085 | 0.072 | 0.028 | -0.032 |
|  | PH | 0.373 | 0.358 | 0.522 | 1.000 | 0.038 | 0.076 | 0.101 | 0.075 | 0.015 | -0.052 | 0.116 | -0.005 |
|  | LP | 0.407 | 0.397 | 0.442 | 0.330 | 1.000 | 0.217 | 0.122 | 0.179 | 0.090 | 0.122 | -0.014 | 0.072 |
|  | W | 0.598 | 0.600 | 0.498 | 0.367 | 0.527 | 1.000 | 0.128 | 0.018 | -0.030 | 0.019 | 0.034 | 0.026 |
|  | PG | 0.321 | 0.294 | 0.323 | 0.309 | 0.430 | 0.378 | 1.000 | 0.328 | 0.118 | -0.021 | 0.010 | 0.033 |
|  | GU | 0.187 | 0.200 | 0.215 | 0.212 | 0.362 | 0.252 | 0.466 | 1.000 | 0.023 | 0.002 | -0.011 | 0.000 |
|  | C | 0.354 | 0.342 | 0.365 | 0.244 | 0.427 | 0.337 | 0.318 | 0.208 | 1.000 | 0.557 | 0.065 | -0.073 |
|  | SR | 0.422 | 0.412 | 0.416 | 0.258 | 0.436 | 0.396 | 0.310 | 0.210 | 0.687 | 1.000 | -0.048 | 0.117 |
|  | E | 0.432 | 0.498 | 0.373 | 0.318 | 0.282 | 0.387 | 0.212 | 0.146 | 0.243 | 0.281 | 1.000 | 0.570 |
|  | WS | 0.405 | 0.467 | 0.323 | 0.269 | 0.296 | 0.368 | 0.220 | 0.149 | 0.224 | 0.297 | 0.714 | 1.000 |
| United Kingdom |  |  |  |  |  |  |  |  |  |  |  |  |  |
|  | HA | 1.000 | 0.520 | 0.196 | -0.012 | 0.022 | 0.199 | 0.099 | 0.011 | 0.030 | 0.041 | 0.005 | -0.007 |
|  | LS | 0.847 | 1.000 | 0.049 | 0.086 | -0.012 | 0.372 | -0.086 | 0.039 | 0.011 | 0.079 | 0.076 | 0.037 |
|  | MH | 0.682 | 0.644 | 1.000 | 0.326 | 0.112 | 0.027 | -0.008 | 0.016 | 0.064 | 0.038 | 0.054 | 0.094 |
|  | PH | 0.450 | 0.468 | 0.524 | 1.000 | 0.046 | -0.004 | 0.073 | 0.049 | -0.028 | -0.008 | -0.010 | 0.026 |
|  | LP | 0.581 | 0.572 | 0.533 | 0.376 | 1.000 | 0.255 | 0.105 | 0.124 | 0.085 | 0.070 | 0.019 | 0.004 |
|  | W | 0.758 | 0.774 | 0.602 | 0.417 | 0.632 | 1.000 | 0.087 | 0.005 | 0.072 | -0.039 | 0.005 | -0.024 |
|  | PG | 0.421 | 0.376 | 0.376 | 0.293 | 0.426 | 0.419 | 1.000 | 0.355 | 0.083 | -0.023 | 0.027 | 0.009 |
|  | GU | 0.384 | 0.368 | 0.343 | 0.303 | 0.413 | 0.386 | 0.484 | 1.000 | 0.020 | 0.024 | -0.027 | -0.009 |
|  | C | 0.606 | 0.598 | 0.534 | 0.326 | 0.558 | 0.583 | 0.403 | 0.348 | 1.000 | 0.718 | -0.024 | 0.014 |
|  | SR | 0.602 | 0.602 | 0.527 | 0.329 | 0.529 | 0.560 | 0.366 | 0.333 | 0.846 | 1.000 | 0.012 | 0.016 |
|  | E | 0.431 | 0.457 | 0.444 | 0.294 | 0.330 | 0.377 | 0.220 | 0.187 | 0.315 | 0.319 | 1.000 | 0.702 |
|  | WS | 0.431 | 0.455 | 0.457 | 0.313 | 0.334 | 0.376 | 0.239 | 0.191 | 0.344 | 0.339 | 0.773 | 1.000 |
| United States |  |  |  |  |  |  |  |  |  |  |  |  |  |
|  | HA | 1.000 | 0.491 | 0.278 | -0.002 | 0.020 | 0.183 | 0.049 | 0.004 | 0.030 | 0.069 | 0.003 | 0.008 |
|  | LS | 0.837 | 1.000 | 0.072 | 0.089 | -0.016 | 0.290 | -0.045 | -0.006 | 0.033 | 0.058 | 0.090 | 0.056 |
|  | MH | 0.737 | 0.682 | 1.000 | 0.211 | 0.141 | 0.064 | 0.081 | 0.053 | 0.087 | -0.028 | 0.073 | -0.021 |
|  | PH | 0.471 | 0.486 | 0.489 | 1.000 | -0.020 | 0.040 | 0.075 | 0.065 | -0.031 | 0.039 | 0.017 | 0.056 |
|  | LP | 0.584 | 0.566 | 0.568 | 0.348 | 1.000 | 0.275 | 0.129 | 0.093 | 0.100 | 0.074 | 0.024 | -0.014 |
|  | W | 0.727 | 0.737 | 0.645 | 0.448 | 0.654 | 1.000 | 0.060 | 0.021 | 0.061 | -0.008 | -0.028 | 0.028 |
|  | PG | 0.424 | 0.379 | 0.430 | 0.297 | 0.445 | 0.435 | 1.000 | 0.353 | 0.032 | 0.006 | -0.020 | 0.013 |
|  | GU | 0.361 | 0.347 | 0.378 | 0.288 | 0.392 | 0.376 | 0.500 | 1.000 | 0.059 | -0.025 | 0.012 | -0.007 |
|  | C | 0.634 | 0.615 | 0.586 | 0.349 | 0.592 | 0.616 | 0.403 | 0.349 | 1.000 | 0.726 | -0.030 | 0.020 |
|  | SR | 0.621 | 0.607 | 0.558 | 0.349 | 0.557 | 0.585 | 0.383 | 0.327 | 0.859 | 1.000 | 0.003 | 0.003 |
|  | E | 0.416 | 0.467 | 0.392 | 0.313 | 0.297 | 0.368 | 0.197 | 0.215 | 0.306 | 0.302 | 1.000 | 0.743 |
|  | WS | 0.404 | 0.450 | 0.375 | 0.303 | 0.291 | 0.368 | 0.204 | 0.204 | 0.310 | 0.302 | 0.780 | 1.000 |
| Sweden |  |  |  |  |  |  |  |  |  |  |  |  |  |
|  | HA | 1.000 | 0.549 | 0.237 | -0.003 | 0.041 | 0.145 | 0.025 | 0.007 | 0.038 | 0.070 | -0.025 | 0.026 |
|  | LS | 0.870 | 1.000 | 0.111 | 0.094 | -0.026 | 0.322 | -0.043 | -0.003 | 0.047 | 0.058 | 0.078 | -0.005 |
|  | MH | 0.728 | 0.709 | 1.000 | 0.178 | 0.090 | 0.066 | 0.083 | -0.027 | 0.022 | 0.018 | 0.100 | 0.023 |
|  | PH | 0.468 | 0.495 | 0.492 | 1.000 | 0.027 | 0.032 | 0.031 | 0.092 | -0.027 | 0.026 | 0.047 | 0.030 |
|  | LP | 0.582 | 0.575 | 0.548 | 0.368 | 1.000 | 0.306 | 0.140 | 0.057 | 0.089 | 0.033 | -0.005 | 0.010 |
|  | W | 0.764 | 0.789 | 0.659 | 0.453 | 0.649 | 1.000 | 0.088 | 0.019 | -0.004 | 0.024 | 0.001 | -0.004 |
|  | PG | 0.402 | 0.391 | 0.402 | 0.293 | 0.427 | 0.432 | 1.000 | 0.292 | 0.081 | -0.007 | -0.020 | 0.038 |
|  | GU | 0.246 | 0.240 | 0.230 | 0.248 | 0.262 | 0.258 | 0.395 | 1.000 | 0.013 | -0.010 | -0.010 | 0.003 |
|  | C | 0.624 | 0.625 | 0.542 | 0.353 | 0.528 | 0.578 | 0.395 | 0.221 | 1.000 | 0.740 | -0.020 | 0.033 |
|  | SR | 0.640 | 0.642 | 0.546 | 0.368 | 0.524 | 0.586 | 0.376 | 0.215 | 0.857 | 1.000 | 0.000 | 0.017 |
|  | E | 0.421 | 0.450 | 0.445 | 0.336 | 0.311 | 0.384 | 0.210 | 0.135 | 0.322 | 0.333 | 1.000 | 0.672 |
|  | WS | 0.417 | 0.430 | 0.424 | 0.322 | 0.312 | 0.378 | 0.231 | 0.147 | 0.341 | 0.346 | 0.750 | 1.000 |
| *Note.* H = happy; LS = life satisfaction; MH = mental health; PH = physical health; SP = sense of purpose; W = life worthwhile; PG = promote good; GU = give up happiness; C = content with relationships; SR = satisfying relationships; E = worry about expenses; WS = worry about safety. | | | | | | | | | | | | | |

**TableS2.** Expected Influence by Country.

| **Country** | H | LS | MH | PH | SP | W | PG | GU | C | SR | E | WS |
| --- | --- | --- | --- | --- | --- | --- | --- | --- | --- | --- | --- | --- |
| Argentina | 1.006 | 1.084 | 0.972 | 0.605 | 0.832 | 0.957 | 0.687 | 0.457 | 0.775 | 0.949 | 0.891 | 0.776 |
| Australia | 1.099 | 1.076 | 1.041 | 0.545 | 0.846 | 0.896 | 0.571 | 0.489 | 1.007 | 1.009 | 0.915 | 0.804 |
| Brazil | 1.009 | 1.127 | 0.958 | 0.618 | 0.866 | 0.999 | 0.718 | 0.531 | 0.971 | 0.959 | 0.870 | 0.699 |
| Egypt | 0.868 | 0.806 | 0.750 | 0.635 | 0.803 | 0.736 | 0.591 | 0.377 | 0.814 | 0.716 | 0.881 | 0.760 |
| Germany | 1.004 | 1.141 | 0.976 | 0.589 | 0.725 | 0.789 | 0.507 | 0.421 | 1.027 | 0.908 | 0.854 | 0.776 |
| Hong Kong | 1.174 | 1.033 | 0.864 | 0.734 | 0.896 | 0.963 | 1.003 | 0.641 | 0.880 | 0.908 | 0.833 | 0.762 |
| India | 0.889 | 1.015 | 0.933 | 0.813 | 0.747 | 0.893 | 0.779 | 0.523 | 0.884 | 0.764 | 0.753 | 0.631 |
| Indonesia | 0.862 | 0.974 | 1.003 | 0.835 | 0.814 | 0.900 | 0.898 | 0.718 | 0.847 | 0.852 | 0.791 | 0.660 |
| Israel | 1.028 | 0.792 | 1.001 | 0.642 | 0.863 | 1.061 | 0.716 | 0.511 | 0.960 | 1.042 | 0.901 | 0.899 |
| Japan | 0.960 | 1.153 | 1.113 | 0.652 | 0.908 | 0.923 | 0.951 | 0.428 | 0.989 | 1.078 | 0.855 | 0.877 |
| Kenya | 0.840 | 0.725 | 0.760 | 0.826 | 0.747 | 0.843 | 0.724 | 0.659 | 0.816 | 0.858 | 0.758 | 0.705 |
| Mexico | 0.906 | 1.136 | 0.981 | 0.614 | 0.795 | 0.912 | 0.798 | 0.492 | 0.949 | 0.941 | 0.809 | 0.753 |
| Nigeria | 0.731 | 0.865 | 0.718 | 0.833 | 0.822 | 0.817 | 0.774 | 0.826 | 0.858 | 0.870 | 0.763 | 0.603 |
| Philippines | 0.847 | 0.955 | 0.888 | 0.794 | 0.875 | 0.734 | 0.879 | 0.803 | 0.869 | 0.903 | 0.687 | 0.672 |
| Poland | 1.105 | 1.034 | 0.875 | 0.716 | 0.803 | 1.005 | 0.764 | 0.317 | 1.056 | 1.013 | 0.856 | 0.901 |
| South Africa | 0.895 | 0.908 | 0.706 | 0.718 | 0.754 | 0.820 | 0.765 | 0.642 | 0.874 | 0.730 | 0.666 | 0.722 |
| Spain | 0.992 | 1.108 | 0.893 | 0.590 | 0.719 | 0.941 | 0.575 | 0.536 | 0.940 | 0.939 | 0.701 | 0.745 |
| Tanzania | 0.903 | 0.675 | 0.702 | 0.906 | 0.803 | 1.001 | 0.807 | 0.574 | 0.892 | 0.738 | 0.883 | 0.826 |
| Türkiye | 0.855 | 1.103 | 0.976 | 0.705 | 0.936 | 0.860 | 0.798 | 0.582 | 0.777 | 0.867 | 0.852 | 0.777 |
| United Kingdom | 1.055 | 1.173 | 0.934 | 0.581 | 0.797 | 0.948 | 0.718 | 0.567 | 1.022 | 0.908 | 0.832 | 0.832 |
| United States | 1.120 | 1.117 | 1.011 | 0.540 | 0.805 | 0.996 | 0.712 | 0.619 | 1.087 | 0.914 | 0.870 | 0.869 |
| Sweden | 1.107 | 1.189 | 0.881 | 0.531 | 0.756 | 0.982 | 0.733 | 0.414 | 1.024 | 0.952 | 0.872 | 0.822 |
| Note. H = happy; LS = life satisfaction; MH = mental health; PH = physical health; SP = sense of purpose; W = life worthwhile; PG = promote good; GU = give up happiness; C = content with relationships; SR = satisfying relationships; E = worry about expenses; WS = worry about safety. | | | | | | | | | | | | |

**TableS3.** Predictability (*R^2^*) by Country.

| **Country** | H | LS | MH | PH | SP | W | PG | GU | C | SR | E | WS |
| --- | --- | --- | --- | --- | --- | --- | --- | --- | --- | --- | --- | --- |
| Argentina | 0.573 | 0.592 | 0.413 | 0.274 | 0.371 | 0.504 | 0.250 | 0.145 | 0.439 | 0.487 | 0.619 | 0.613 |
| Australia | 0.741 | 0.730 | 0.645 | 0.271 | 0.506 | 0.607 | 0.237 | 0.165 | 0.773 | 0.770 | 0.611 | 0.588 |
| Brazil | 0.573 | 0.592 | 0.413 | 0.274 | 0.371 | 0.504 | 0.250 | 0.145 | 0.439 | 0.487 | 0.619 | 0.613 |
| Egypt | 0.260 | 0.245 | 0.170 | 0.159 | 0.199 | 0.206 | 0.122 | 0.062 | 0.273 | 0.258 | 0.390 | 0.377 |
| Germany | 0.682 | 0.710 | 0.440 | 0.324 | 0.282 | 0.476 | 0.143 | 0.098 | 0.644 | 0.641 | 0.541 | 0.529 |
| Hong Kong | 0.647 | 0.604 | 0.485 | 0.424 | 0.447 | 0.547 | 0.515 | 0.339 | 0.519 | 0.531 | 0.486 | 0.460 |
| India | 0.387 | 0.432 | 0.385 | 0.341 | 0.248 | 0.338 | 0.264 | 0.140 | 0.351 | 0.326 | 0.360 | 0.346 |
| Indonesia | 0.391 | 0.428 | 0.410 | 0.362 | 0.340 | 0.397 | 0.373 | 0.305 | 0.394 | 0.393 | 0.413 | 0.394 |
| Israel | 0.534 | 0.425 | 0.458 | 0.332 | 0.411 | 0.512 | 0.291 | 0.203 | 0.699 | 0.696 | 0.590 | 0.579 |
| Japan | 0.775 | 0.799 | 0.717 | 0.518 | 0.607 | 0.688 | 0.613 | 0.246 | 0.792 | 0.804 | 0.645 | 0.653 |
| Kenya | 0.236 | 0.224 | 0.234 | 0.245 | 0.212 | 0.235 | 0.205 | 0.183 | 0.270 | 0.266 | 0.330 | 0.319 |
| Mexico | 0.545 | 0.606 | 0.452 | 0.302 | 0.366 | 0.504 | 0.331 | 0.187 | 0.503 | 0.497 | 0.574 | 0.573 |
| Nigeria | 0.256 | 0.314 | 0.296 | 0.307 | 0.290 | 0.260 | 0.329 | 0.348 | 0.369 | 0.364 | 0.408 | 0.388 |
| Philippines | 0.360 | 0.382 | 0.354 | 0.317 | 0.353 | 0.274 | 0.380 | 0.366 | 0.414 | 0.416 | 0.360 | 0.359 |
| Poland | 0.661 | 0.652 | 0.551 | 0.469 | 0.443 | 0.576 | 0.373 | 0.152 | 0.668 | 0.663 | 0.632 | 0.636 |
| South Africa | 0.285 | 0.327 | 0.240 | 0.244 | 0.227 | 0.270 | 0.242 | 0.213 | 0.337 | 0.314 | 0.378 | 0.379 |
| Spain | 0.670 | 0.689 | 0.376 | 0.283 | 0.282 | 0.608 | 0.207 | 0.171 | 0.527 | 0.543 | 0.502 | 0.501 |
| Tanzania | 0.325 | 0.282 | 0.248 | 0.309 | 0.255 | 0.337 | 0.254 | 0.172 | 0.328 | 0.291 | 0.377 | 0.367 |
| Türkiye | 0.534 | 0.580 | 0.420 | 0.290 | 0.348 | 0.393 | 0.293 | 0.232 | 0.426 | 0.434 | 0.475 | 0.454 |
| United Kingdom | 0.700 | 0.732 | 0.489 | 0.300 | 0.413 | 0.634 | 0.313 | 0.246 | 0.675 | 0.668 | 0.579 | 0.576 |
| United States | 0.771 | 0.766 | 0.636 | 0.323 | 0.543 | 0.690 | 0.376 | 0.321 | 0.769 | 0.752 | 0.654 | 0.649 |
| Sweden | 0.785 | 0.799 | 0.588 | 0.283 | 0.480 | 0.685 | 0.322 | 0.151 | 0.753 | 0.752 | 0.577 | 0.560 |
| Note. H = happy; LS = life satisfaction; MH = mental health; PH = physical health; SP = sense of purpose; W = life worthwhile; PG = promote good; GU = give up happiness; C = content with relationships; SR = satisfying relationships; E = worry about expenses; WS = worry about safety. | | | | | | | | | | | | |
